# Biomass based carbon quantum dots in sensor applications: a review (2021–2025)

**DOI:** 10.1186/s40643-026-01084-7

**Published:** 2026-06-08

**Authors:** Elyor Berdimurodov, Khasan Berdimuradov, Ashish Kumar, Abhinay Thakur, Kamila Rashidova, Jasur Tursunqulov, Khudaybergan Polvonov, Alisher Ishankulov, Nafosat Oramova, Ahmad Hosseini-Bandegharaei, Bobirmirzo Khasanov, Rasulbek Eshmetov

**Affiliations:** 1 Faculty of Chemistry, National University of Uzbekistan, 100034 Tashkent, Uzbekistan; 2 University of Tashkent for Applied Sciences, Str. Gavhar 1, 100149 Tashkent, Uzbekistan; 3 School of Medicine, Central Asian University, 111221 Tashkent, Uzbekistan; 5 Department of Pharmacy and Chemistry, Alfraganus University, 100190 Tashkent, Uzbekistan; 6 Chair of Inorganic chemistry, Karshi State University, 180119 Karshi, Uzbekistan; 4 Shahrisabz Faculty of Food Engineering, Karshi State Technical University, Karshi, Uzbekistan; 7 Science Technology and Technical Education Department, Nalanda College of Engineering, Bihar Engineering University, Government of Bihar, Bihar city, 803108 India; 8 Division of Research and Development, Lovely Professional University, Phagwara, Punjab 144411 India; 9 Jizzakh State Pedagogical University, Jizzakh, Uzbekistan; 10 Department of Pharmaceutical and Chemistry, Alfraganus University, 100190 Tashkent, Uzbekistan; 11 Physics and Chemistry, Western Caspian University, AZ-1001 Baku, Azerbaijan; 12 Natural and Agricultural Sciences, Urgench State University named after Abu Rayhan Biruni, 220100 Urgench city, Uzbekistan; 13 Kimyo International University in Tashkent Branch Samarkand, Samarkand, Uzbekistan; 14 University of Economics and Pedagogy, Karshi City, Uzbekistan; 15 Faculty of Chemistry, Semnan University, Semnan, Iran; 16 Scientific Research Center, Al-Ayen Iraqi University (AUIQ), Nasiriyah, Thi-Qar 64001 Iraq; 17 Department of Sustainable Engineering, Saveetha School of Engineering, Saveetha Institute of Medical and Technical Science (SIMATS), Chennai, Tamil Nadu 602105 India; 18 Department of Mechanical Technology, Andijan State Technical Institute, Andijan, Uzbekistan; 19 Natural Sciences, Ma’mun Universiteti, Urgench, Uzbekistan

**Keywords:** Carbon quantum dots, Biomass-derived nanomaterials, Fluorescent sensors, Environmental monitoring, Biological sensing, Metal ion detection

## Abstract

The past few years have witnessed an exponential rise in the development of carbon quantum dots (CQDs) derived from natural biomasses, rendering them a green and multifunctional platform for sensor applications. This comprehensive review provides a critical assessment of advancements between 2021 and 2025 in the syntheses, physicochemical characterizations, and sensing applications of biomass-derived CQDs. Different precursors—e.g., fruit peels, leaves, and agricultural waste—have been effectively transformed into highly fluorescent CQDs using hydrothermal, microwave-assisted, and pyrolytic routes, with synthesis times as short as 10 min for certain microwave procedures. The structural studies exhibit quasi-spherical morphologies (2–10 nm) and partial graphitization, and the optical studies confirm excitation-dependent fluorescence with quantum yields as high as 30%, particularly in nitrogen- and sulfur-doped systems. Biomass-derived CQDs have shown superb selectivity and sensitivity to a broad spectrum of analytes. For example, CQDs synthesized from Solanum nigrum leaves reported detection limits as low as 8 nM for Fe³⁺, while CQDs synthesized from pitaya peels enabled sensitive detection of antibiotics via aggregation-induced emission effects. Environmental targets such as glyphosate, NH₃, and CH₂O have also been detected at nanomolar levels, employing mechanisms like static/dynamic fluorescence quenching, electron transfer, and FRET. Despite these advances, there are still challenges in large-scale production, standardization of fabrication protocols, and integration of CQDs into real-time sensing platforms. In the future, the work is going to be further developed through the adoption of green synthesis approaches, regulatory standardizations, and integration of CQDs into wearable and portable diagnostic devices. Such developments point to the prospects of biomass-sourced CQDs as green, cost-effective, and ultrasensitive nanomaterials for next-generation chemical, biological, and environmental sensors.

**Graphical abstract:**

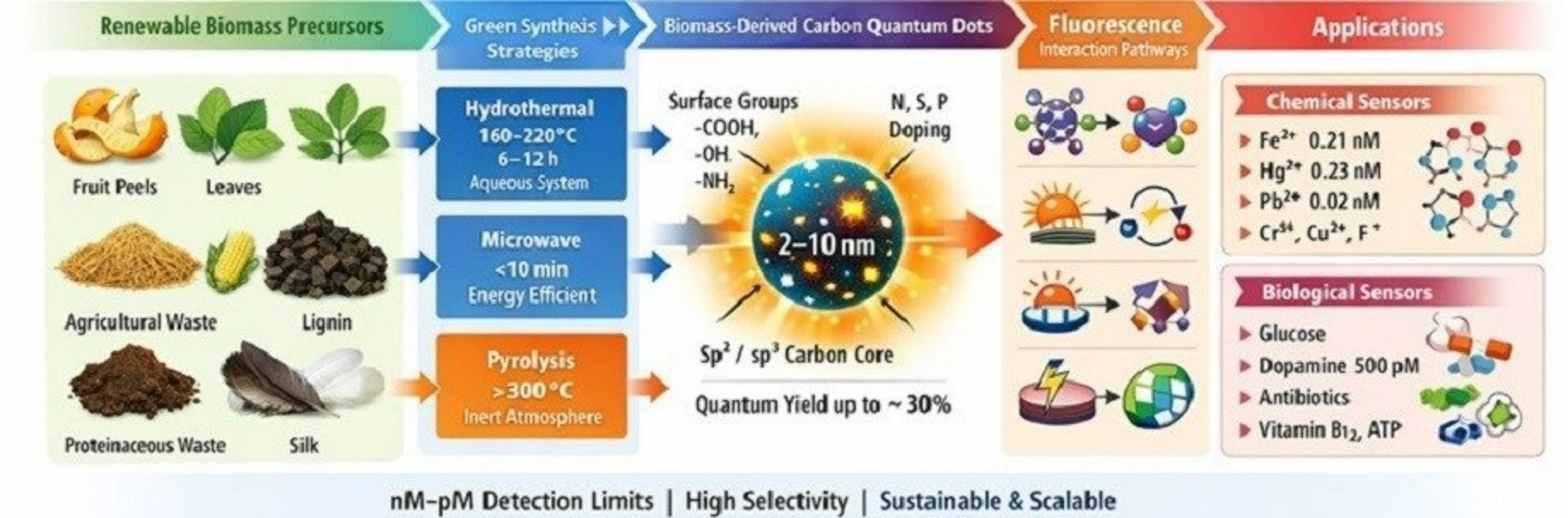

## Introduction

Carbon Quantum Dots (CQDs) are a class of zero-dimensional, carbon-based nanomaterials typically below 10 nm in size, known for their extraordinary fluorescence, biocompatibility, tunable optical properties, and chemical inertness. These attributes make them highly suitable for exploitations in chemical, biological, and environmental sensing (Ahmed and Soylak 2024, Al-Ghamdi et al. 2023). Unlike traditional semiconductor quantum dots, CQDs demonstrate low toxicities and are easily dispersible in waters, owing to the presence of copious oxygen- and nitrogen-containing functional moieties on the surfaces (Alhazzani et al. 2023, Alomar et al. 2024). Their unique excitation-dependent fluorescence and good photostability allow for real-time and label-free detection of analytes through mechanisms such as fluorescence quenching, energy transferring, and surface complexation. The adaptability of their surface chemistry through functionalization enhances their sensitivity and selectivity toward specific targets, which has led to a surge in interest for their integration into sensor technologies, particularly for metal ion detection, pH sensing, drug monitoring, and pollutant tracking (Ayiloor Rajesh et al. 2022, Chen et al. 2022).

In recent years, the development of CQDs from natural biomass has garnered increasing attention due to its sustainability and alignment with green chemistry principles. Biomass-derived CQDs leverage readily available resources like fruits peels, plants leaves, agricultural residues, and biopolymers (cellulose, lignin, etc.), making the synthesis cost-effective and environmentally friendly (Elizabeth et al. 2023, Guan et al. 2022). Compared to conventional procedures that often rely on toxic precursors and harsh conditions, biomass routes offer a cleaner alternative with reduced carbon footprint. Moreover, the natural precursors often give rise to additional functional groups to the CQDs, enhancing their sensing performance by providing specific binding sites for analytes (Gulati et al. 2023, Hu et al. 2024). The high carbon content and inherent heteroatoms in biomass not only contribute to fluorescence but also support doping strategies (e.g., N, S, P), which further tune the electronic and optical properties of CQDs. This sustainable approach ensures scalability and bio-compatibility, especially important for sensors used in biological and environmental fields (Kolaprath et al. 2023, Kundu et al. 2023, Li and Ma 2024).

Despite the increased interest and rapid progress in CQDs from biomass precursors, the area is highly heterogenous with various disparate biomass sources, synthesis methods, and sensing applications reported in dispersed sporadic research studies (Teli et al. 2025, Pechnikova et al. 2025, Yang et al. 2024). This heterogeneity creates highly daunting challenges for investigators to identify optimal synthesis methods, structure–property correlations, and benchmark sensing performance. Further, this domain has undergone rapid evolution from 2021 to 2025, and thus such novel functionalization techniques, doping strategies, and integration approaches need proper systematic consolidation. A significant review should be carried out for the more accurate compilation of up-to-date knowledge for critically assessment of the state-of-the art, explanation of underlying mechanisms governing CQD fluorescence, and interactions with analytes as well as comparison with non-biomass-derived counterparts. This evaluation not only facilitates the logical design of CQD sensors to come but also uncovers present limitations—chiefly in scalability, reproducibility, and integration within devices—that must be addressed for these materials to progress beyond the laboratory curiosity to practical sensing platforms. Through the incorporation of recent developments, performance indices, and emerging trends, the review hopes to provide a useful reference for technologists and researchers who want to harness the full potential of CQDs derived from biomass in cost-effective, sensitive, and sustainable sensor technologies.

Biomass-derived nanocarbons, including CQDs, have emerged as sustainable alternatives to conventional nanomaterials in biosensing due to their renewable sources, tunable surface chemistry, and eco-friendly synthesis. They enable sensitive detection of diverse analytes through fluorescence, electrochemical, and colorimetric platforms (Sharma et al. 2021). Recent reviews highlight their role in medical diagnostics and environmental monitoring (Das et al. 2024), emphasize their contribution to circular bioeconomy models (Tripathi et al. 2023), and demonstrate their applicability in pollutant detection and biocompatible sensing strategies (Kaushik et al. 2022). These advances reinforce biomass-derived nanocarbons as sustainable, high-performance candidates for next-generation biosensors.

Despite the rapid proliferation of reviews on carbon quantum dots in recent years, the existing literature predominantly adopts either a broad focus on CQD synthesis and properties irrespective of precursor origin (Teli et al. 2025, Mathew and Mathew 2023), a narrow emphasis on biomedical applications alone (Pechnikova et al. 2025, Zhang et al. 2022), or a generalized survey of environmental sensing without systematic integration across analyte classes (Yang et al. 2024). While these contributions have substantially advanced the field, a critical gap remains: no review to date has provided a integrated, application-oriented synthesis specifically focused on biomass-derived CQDs developed within the most recent 2021–2025 period, with explicit coverage across chemical, biological, and environmental sensing domains. The present work addresses this gap by offering three distinct contributions. First, we confine our scope exclusively to biomass-derived precursors, emphasizing the green chemistry principles and sustainability advantages that distinguish these materials from conventionally synthesized CQDs. Second, we concentrate on the 2021–2025 timeframe, capturing the most recent advances in synthesis optimization, doping strategies, and functionalization approaches that have dramatically enhanced sensing performance. Third, and most importantly, we introduce a unifying structure–mechanism–performance framework that systematically links precursor selection and synthesis parameters to structural features, dominant photophysical mechanisms, and ultimately to analytical figures of merit across chemical, biological, and environmental applications. This framework provides an analytical lens absent from prior reviews, transforming the work from a literature catalog into a conceptual synthesis that offers researchers a roadmap for the rational design of high-performance, sustainable CQD-based sensors. By explicitly contrasting our focused scope and integrative approach with the broader or more specialized treatments in existing reviews, we aim to establish the unique value proposition of this work and its contribution to advancing the field (Eliboev et al. 2025, Allambergenova et al. 2025 and Kiryigitova et al. 2026).

**Fig. 1:**
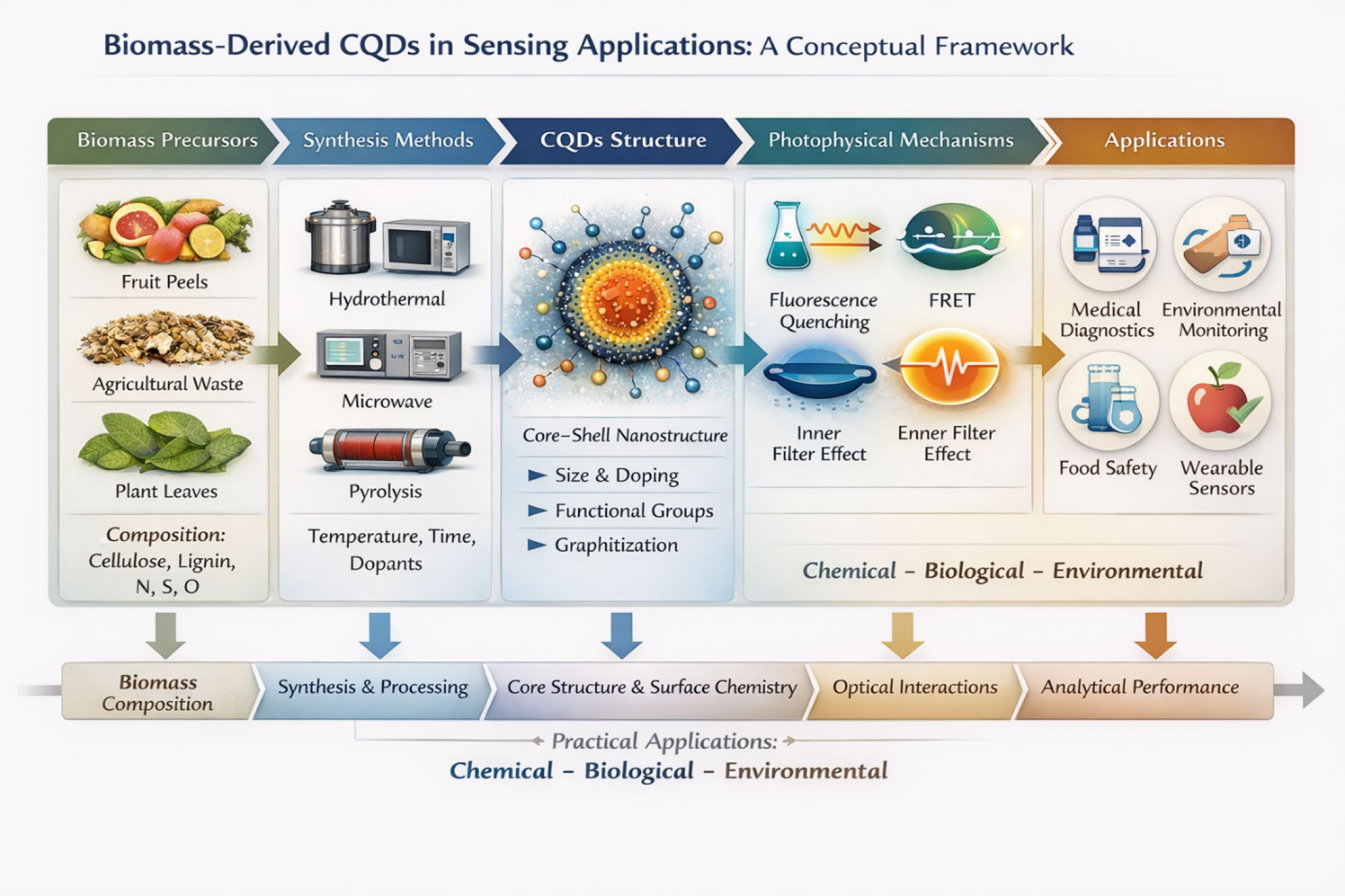
Conceptual structure–mechanism–performance framework for biomass-derived carbon quantum dots (CQDs), illustrating how precursor composition and synthesis parameters determine structural features that govern sensing mechanisms and analytical performance

The current work provides a comprehensive overview of biomass-based carbon quantum dots developed between 2021 and 2025, with a particular focus on their applications in sensor technologies. Emphasis is placed on the use of CQDs as fluorescent probes for chemical (e.g., Fe³⁺, Hg²⁺, Pb²⁺ ions; Cl⁻, NO₃⁻ anions), biological (e.g., glucose, dopamine, antibiotics, pathogens), and environmental sensing (e.g., pesticides, pharmaceuticals, gases like NH₃ and CH₂O). The reviewed literature primarily comprises peer-reviewed research articles and authoritative reviews published in international scientific journals. Given the focus on the recent 2021–2025 period, articles available in ‘early access’ form were considered equivalent to formally published papers, while non-peer-reviewed preprints were excluded to ensure the reliability of the synthesized data. The scope encompasses the synthesis methods from diverse biomass sources, physicochemical characterizations, sensing mechanisms (e.g., static quenching, FRET, inner filter effect), and performance metrics such as detection limit (LOD), linear range, and selectivity. However, unlike previous discussions that often treat synthesis, structure, sensing mechanism, and analytical performance as isolated aspects, this review integrates these elements within a unified structure–mechanism–performance framework (Fig. 1). By highlighting how precursor composition and synthesis parameters govern structural features, which in turn dictate photophysical interactions and ultimately determine sensing efficiency, we aim to present a holistic understanding of the field. Furthermore, the review presents a comparative analysis with non-biomass-derived CQDs, evaluates challenges in scalability and reproducibility, and discusses emerging trends such as integration with wearable devices and real-time monitoring platforms. The objective is to highlight the innovations, efficacy, and versatility of biomass-based CQDs in developing low-cost, eco-friendly, and sensitive sensor systems suitable for various analytical applications.

### Review methodology

To ensure a comprehensive and transparent synthesis of the literature, a systematic search strategy was employed. The following electronic databases were searched for relevant articles published between January 2021 and December 2025: Scopus, Web of Science, PubMed, and Google Scholar. The search utilized a combination of keywords, including: “biomass-derived carbon quantum dots,” “CQDs,” “fluorescent sensor,” “chemical sensor,” “biosensor,” “environmental sensor,” “metal ion detection,” “pesticide detection,” and “heteroatom doping.” The initial search yielded 417 records after duplicate removal. The reference lists of retrieved articles were also manually screened to identify additional relevant studies, though no new records meeting the inclusion criteria were found.

The search was restricted to peer-reviewed research articles and authoritative review papers published in reputable international journals. Conference abstracts, non-peer-reviewed preprints, and studies with insufficient characterization data (e.g., lacking basic fluorescence or structural data) were excluded to maintain the scientific rigor of the synthesized findings. Title and abstract screening eliminated 252 records that were off‑topic, non‑peer‑reviewed, or lacked sufficient characterization. The remaining 131 full‑text articles were assessed for eligibility; 25 were excluded because they did not report clear analytical figures of merit (e.g., limit of detection, linear range). Consequently, 106 articles were finally retained for detailed analysis and synthesis in this review.

## A structure–mechanism–performance framework for the rational design of biomass-derived CQDs

The sensing efficacy of biomass-derived carbon quantum dots (CQDs) is not a function of isolated variables but rather emerges from a deterministic cascade linking precursor chemistry, synthetic conditions, resultant nanoscale architecture, and photophysical behavior. As conceptualized in Fig. 1, this framework establishes a direct structure–mechanism–performance pathway to guide rational materials design. The cascade initiates with biomass selection, where the molecular composition, specifically the carbon framework, lignocellulosic matrix, and intrinsic heteroatom content (e.g., N, S, P), dictates carbonization kinetics and heteroatom self-doping efficiency during synthesis. Key reaction parameters, including hydrothermal temperature, residence time, system pH, and exogenous dopant concentration, subsequently govern nucleation dynamics, the extent of sp²-conjugated domain formation (graphitization), surface defect density, and the speciation of surface functional groups (e.g., carboxyl, hydroxyl, amine).

These synthesis-defined structural attributes altogether determine the electronic band structure and surface-state distribution of the CQDs. The resulting density of states and surface chemistry, in turn, orchestrate the dominant photophysical interactions with target analytes. These include static and dynamic quenching via ground-state complexation or collisional deactivation, photoinduced electron transfer (PET), Förster resonance energy transfer (FRET), and the inner filter effect (IFE).

Indeed, the analytical figures of merit, i.e. sensitivity (quantified by limits of detection), selectivity coefficients, Stern–Volmer constants, and linear dynamic range, are direct, quantifiable manifestations of these underlying structure–mechanism relationships. This integrated framework transcends empirical, trial-and-error synthesis by establishing predictive correlations between tunable input parameters and targeted output performance. By systematically manipulating precursor selection and synthetic variables, it becomes feasible to engineer CQDs with bespoke optical responses optimized for specific chemical, biological, or environmental sensing modalities .

The practical utility of this framework is demonstrated by tracing specific examples from the recent literature (summarized in Table 1). For instance, the lignin-rich composition and aromatic structure of walnut shell precursors promote the formation of graphitic domains and π-rich surfaces during hydrothermal synthesis. These structural features directly enable an excited-state intramolecular proton transfer (ESIPT) mechanism with the insecticide imidacloprid, resulting in a fluorescence “turn-on” response with a detection limit of 7.58 µM (Li et al. 2021). In contrast, the proteinaceous nature of chicken feathers provides intrinsic nitrogen doping, yielding CQDs with amine-rich surfaces. This chemical feature facilitates strong coordination with Hg²⁺ ions, driving an efficient static quenching mechanism and an ultralow detection limit of 6.2 nM (Lv et al. 2022). Similarly, careful manipulation of synthesis parameters (e.g., doping, temperature) allows the same lignin precursor to be tuned for electron transfer mechanisms, achieving sensitive detection of Fe³⁺ and Co²⁺ (Lv et al. 2023). These examples move the framework from a conceptual proposal to an empirically validated roadmap, showing how precursor selection and synthesis conditions can be rationally chosen to engineer the structural features that govern specific sensing mechanisms and, ultimately, analytical performance.

**Table 1:** Empirical validation of the structure–mechanism–performance framework: representative examples of biomass-derived CQDs illustrating the causal cascade from precursor chemistry to analytical performance

Biomass precursor (chemistry type)	Key structural feature (from synthesis)	Dominant sensing mechanism	Target analyte	Performance (LOD)	Ref.
Walnut shell (lignin-rich)	Graphitic domains, π-rich surfaces, –COOH/–OH/–NH₂ groups	ESIPT + π–π stacking → Fluorescence enhancement (“turn-on”)	Imidacloprid (insecticide)	7.58 µM	(Li et al. 2021)
Chicken feather (proteinaceous)	N-doped, amine-rich surfaces, defect-rich	Coordination-based static quenching (“turn-off”)	Hg²⁺	6.2 nM	(Lv et al. 2022)
Banana petiole (cellulosic)	O-rich groups (–OH, –COOH), amorphous carbon	Surface state stabilization → Fluorescence enhancement (“turn-on”)	Fe³⁺	0.21 nM	(Zhu et al. 2021)
Lignin (aromatic polymer)	N-doped, enhanced electron density, extended sp² domains	Photoinduced electron transfer (PET) + Static quenching	Fe³⁺/Co²⁺	0.45 µM / 0.50 µM	(Lv et al. 2023)
Pitaya peel (hybrid)	N-doped, surface oxidation, -COOH/-NH₂	AIE + IFE + Static quenching	Tetracyclines	33.8 nM (TC)	(Pang et al. 2022)

## Synthesis of biomass-based carbon quantum dots

Biomass-derived CQDs are synthesized utilizing a broad spectrum of natural sources, offering abundant, renewable, and low-cost precursors. Common biomass feedstocks include plant leaves (e.g., Solanum nigrum, Ziziphus mauritiana, and Artemisia annua), fruit peels (e.g., orange, lemon, banana, and pitaya), and agricultural waste products (e.g., rice husk, corn cob, and wheat straw) (Dhandapani et al. 2023, Chen et al. 2024, Manikandan and Min 2023). These organic materials are rich in carbon, hydrogen, oxygen, and heteroatoms like nitrogen or sulfur, which contribute to inherent fluorescence properties and facilitate heteroatom doping during synthesis. The structural diversity and varying composition of these precursors allow tuning of CQD properties such as size, emission wavelength, and functional group content, thus optimizing them for specific sensor applications.

Beyond categorization by source type, biomass precursors are more meaningfully distinguished by their predominant chemical constituents, namely cellulosic, lignin-rich, and proteinaceous compositions, each of which imparts distinct structural and optical characteristics to the resulting CQDs. Cellulosic biomass (e.g., fruit peels, straw, corn cob) is rich in polysaccharides that undergo dehydration and carbonization to yield CQDs with abundant oxygen-containing functional groups (C=O, C–O, O–H) and predominantly amorphous carbon cores (Dhandapani et al. 2023, Chen et al. 2024). These surface oxides facilitate hydrophilic stability and metal ion coordination but may limit graphitic crystallinity. Lignin-rich precursors (e.g., walnut shell, alkali lignin, woody tissues) contain aromatic polymer networks that promote the formation of larger sp²-conjugated domains during carbonization, resulting in CQDs with enhanced graphitic character and red-shifted emission (Lv et al. 2023, Mansi and Gaurav 2023). The preservation of aromatic subunits contributes to higher photostability and π–π interaction capabilities with aromatic analytes. Proteinaceous biomass (e.g., chicken feathers, silk, eggshell membrane) introduces intrinsic nitrogen doping through amino acid decomposition, generating CQDs with amine-rich surfaces and n→π* transitions that enhance quantum yield and enable distinct coordination chemistry with metal ions (Lv et al. 2022, Nagaraj et al. 2022). Some precursors, such as spent tea leaves or moringa oleifera, exhibit hybrid compositions (lignocellulosic + proteinaceous), yielding CQDs with combined features. This chemistry-driven classification is not merely taxonomic; it directly informs the resulting CQD core structure (amorphous vs. graphitic), surface functionality (oxygen-rich vs. nitrogen-rich), and emissive state distribution, which together determine sensing performance for specific analyte classes. This precursor chemistry–structure relationship forms the foundation of the structure–mechanism–performance framework illustrated in Fig. 1. By understanding how biomass composition dictates carbonization pathways and surface chemistry, researchers can rationally select precursors to target desired CQD properties for specific sensing applications.

Furthermore, beyond the choice of precursor, synthesis parameters such as temperature, reaction time, and precursor heteroatom content directly regulate particle size, degree of graphitization, and surface functionalization. These factors are decisive in determining the fluorescence behavior and, consequently, the overall sensing performance of the resulting CQDs.

### Hydrothermal and solvothermal techniques

Among the most widely used methods for biomass CQD synthesis is the hydrothermal process, which involves heating a biomass slurry in a sealed Teflon-lined autoclave at elevated temperatures (typically 160–220 °C) for several hours (commonly 6–12 h). During this process, biomass undergoes dehydration, carbonization, and passivation, resulting in nanosized fluorescent carbon dots. This method is simple, reproducible, and effective in preserving functional groups that are essential for fluorescence and sensing (Osman et al. 2024, Pechnikova et al. 2025). For instance, Solanum nigrum leaf powder subjected to hydrothermal treatment yielded CQDs with strong optical response suitable for phloroglucinol sensing. Similarly, solvothermal synthesis, which uses organic solvents instead of water, can enhance carbonization and doping efficiency, especially for less water-soluble biomass sources.

### Microwave-assisted synthesis

The microwave method is a fast and energy-efficient alternative that accelerates carbonization and nucleation through uniform microwave heating. This technique typically requires only a few minutes, significantly reducing synthesis time. For example, CQDs derived from orange peel and ginkgo kernel were synthesized in under 10 min using microwave irradiation, showing excellent fluorescence suitable for sensing nitrite and nitric oxide (Preethi et al. 2022, Raikwar 2022). Microwave-assisted methods are particularly advantageous for scale-up due to their simplicity and shorter reaction cycles.

### Pyrolysis and carbonization

Pyrolysis involves heating the biomass precursor at high temperatures (typically > 300 °C) under an inert atmosphere (e.g., N_2_ or Ar) to induce thermal decomposition and carbon core formation. Carbonization results in the breakdown of long-chain organics and formation of nanoscale sp² clusters that exhibit quantum confinement. For instance, walnut shell powder and loquat fruit pulp were subjected to pyrolysis to synthesize CQDs with excellent pollutant-sensing capabilities such as for imidacloprid and permanganate ions (Selvaraju et al. 2022, Sun et al. 2021). Though more energy-intensive, this method allows for high carbon yield and control over core graphitization.

### Surface functionalization

Post-synthesis modification of CQDs is critical to enhance their selectivity, dispersibility, and analyte-binding affinity. Biomass-derived CQDs often retain oxygen-rich groups (–OH, –COOH) and nitrogen functionalities (–NH_2_) from the natural precursors, which already contribute to their sensing abilities. However, targeted surface passivation using polyethylene glycol (PEG), amines, or thiol-containing compounds further improves fluorescence quantum yield and biocompatibility (Vyas et al. 2023, Yang et al. 2024). N, S, and P dopants modulate the electronic structure, enhance conductivity, and improve fluorescence. Ligands or recognition elements (e.g., aptamers, amino acids) can be attached to enhance molecular recognition. Integration with metal-organic frameworks (MOFs), polymers, or other nanomaterials like ZnO or AuNPs boosts the CQDs’ sensitivity and stability under harsh conditions. An excellent example is the CQDs derived from Moringa oleifera, where nitrogen doping and appropriate surface states enabled highly selective detection of H₂O₂ and aspartic acid through FRET-based mechanisms (Yong et al. 2023, Zhang et al. 2024). Similarly, functionalized pitaya peel-based CQDs demonstrated aggregation-induced emission (AIE) effects, increasing the signal response toward antibiotics like chlortetracycline.

It should be mentioned that the synthesis parameters collectively determine particle size, degree of graphitization, defect density, and surface functional groups, which directly influence the optical properties and sensing mechanisms of the resulting CQDs. Also, while these methods are widely adopted for their simplicity and effectiveness, the energy demands and environmental footprints of each approach vary considerably and are discussed further in Sect.  8.

## Physicochemical properties of biomass-based CQDs

Understanding the physicochemical features of biomass-derived CQDs is essential for tailoring their performance in sensing exploitations. Such properties—governed by the choice of biomass, fabrication route, and post-treatment—include size and morphology, crystalline structure, optical characteristics, and surface chemical composition. Each of mentioned factors has a critical role in dictating the CQDs’ fluorescence behavior, chemical reactivity, and interaction with analytes.

### Structural characteristics

Biomass-derived CQDs typically exhibit spherical morphology with sizes spanning from 2 to 10 nm, as confirmed by transmission electron microscopy (TEM). High-resolution TEM (HRTEM) evaluations often reveal well-dispersed particles with lattice fringes indicating partial graphitization, suggesting the presence of sp²-hybridized carbon domains. For example, CQDs synthesized from Solanum nigrum and loquat fruit showed lattice spacing around 0.21–0.24 nm, assignable to (100) and (002) planes of graphitic carbon, indicating semi-crystalline nature. In some cases, the carbon cores are embedded in an amorphous matrix, especially when derived from complex precursors like lignocellulose or protein-rich waste. X-ray diffraction (XRD) patterns usually display broad humps centered around 20–25°, confirming the amorphous or turbostratic nature of carbon in most biomass CQDs. This structural variability affects quantum confinement and thereby modulates their fluorescence efficiency (Zhang et al. 2023, Zhang et al. 2023).

### Optical properties

 The standout feature of CQDs is their excitation-dependent fluorescence, yet the precise origin of this behavior remains an active subject of debate in the literature. Three competing mechanisms have been proposed: (i) size distribution effects, where quantum confinement in sp² domains of varying dimensions produces different bandgaps and thus emission wavelengths; (ii) surface state-mediated emission, where functional groups (C=O, C–OH, C–N) introduce discrete energy levels that dominate photoluminescence independent of core size; and (iii) molecular fluorophores, where small fluorescent molecules formed during carbonization attach to the CQD surface or exist freely in solution, contributing to observed emission (Teli et al. 2025, Zhang et al. 2022). Each mechanism has received some experimental support, though the evidence is often indirect: TEM reveals size polydispersity (2–10 nm), which has been interpreted as consistent with quantum confinement, while FTIR and XPS confirm abundant surface functional groups that have been shown to correlate with emission shifts. Some studies attribute excitation-dependent behavior primarily to surface oxidation states, noting that reduction or passivation alters fluorescence without changing particle size (Zhang et al. 2022). Others suggest that molecular fluorophores formed from precursor decomposition may dominate, particularly in low-temperature syntheses (Teli et al. 2025). Given the current state of knowledge, multiple mechanisms may operate simultaneously in many cases, with their relative contributions likely depending on precursor chemistry and synthesis conditions. Nevertheless, direct experimental confirmation of the dominant pathway in a given CQD system remains challenging, and interpretations should be made with appropriate caution. UV-Vis absorption spectra typically display strong peaks below 300 nm associated with π→π* transitions of aromatic C=C bonds and shoulders or tails extending beyond 350 nm assignable to n→π* transitions of C=O groups, observations that are consistent with both core and surface contributions. For example, Ziziphus mauritiana-derived CQDs exhibited absorption near 280 nm with visible fluorescence underneath UV light, a finding suggestive of surface defect states responsible for green or blue emission (Zhou et al. 2023, Hao et al. 2023).

Photoluminescence (PL) emission spectra show variable emission wavelengths depending on the excitation source, a property exploited for multicolor imaging and ratiometric sensing. Biomass CQDs synthesized from orange peel, cherry tomato stalk, and banana flower bracts have demonstrated fluorescence emissions spanning 420–550 nm, tunable with excitation between 320 and 460 nm. The quantum yield (QY) of biomass CQDs ranges from 5% to over 30%, influenced by heteroatom doping, surface passivation, and purification. Higher QYs are typically observed when nitrogen or sulfur dopants are introduced, as in the case of Moringa oleifera and banana stem-derived CQDs. Notably, reported QY values must be interpreted with caution due to inconsistencies in measurement protocols across studies. Most determinations use relative methods comparing integrated photoluminescence intensity against a reference standard (e.g., quinine sulfate in 0.1 M H₂SO₄, QY = 54%), but variations in excitation wavelength, choice of reference standard, solvent refractive index correction, and instrument calibration can introduce significant discrepancies (Teli et al. 2025). These methodological differences complicate direct comparison of QY values reported from different laboratories and highlight the need for standardized reporting practices. Photostability is another key property for sensor reliability (Zhang et al. 2022). Many biomass CQDs retain stable fluorescence over long-term exposure to UV light and resist photobleaching—particularly those embedded in polymeric matrices or containing antioxidative functional groups such as polyphenols (e.g., in tea leaf- or gooseberry-derived CQDs).

Hydroxyl (–OH), carboxyl (–COOH), carbonyl (C=O), and amine (–NH_2_) functional groups are responsible for:

Hydrogen bonding and electrostatic interaction with analytes. Fluorescence quenching or enhancement, particularly in the presence of metal ions or electron acceptors (Ding et al. 2021, Li et al. 2024). For example, CQDs derived from pitaya peel, purslane, and rice husk exhibited abundant surface functional groups that enhanced their selectivity for tetracycline, formaldehyde, and fluoroquinolones, respectively.

The physicochemical properties of biomass-based CQDs—defined by their nanometric size, structural arrangement, tunable fluorescence, and diverse surface chemistry—are instrumental in determining their performance in sensor platforms. Their small, quasi-spherical morphology and crystalline domains enable quantum confinement, while functional groups and dopants impart selectivity and stability. These synergistic features make biomass CQDs ideal for highly sensitive detection across chemical, biological, and environmental domains. Therefore, structural features established during synthesis, particularly surface states and heteroatom doping, play a decisive role in defining the dominant fluorescence response pathways exploited in sensing applications.

## Sensor applications

In selecting suitable sensing platforms, CQDs stand out compared to existing sensors due to their combination of eco-friendly synthesis, low toxicity, and tunable fluorescence. Unlike conventional semiconductor quantum dots or metallic nanoparticles that often raise safety and cost concerns, biomass-derived CQDs are renewable, biocompatible, and photostable, making them attractive for biological and environmental applications. Their abundant surface functionalities enable easy modification, offering competitive sensitivity and selectivity that in many cases matches or surpasses traditional optical and electrochemical sensors. These attributes highlight CQDs as a versatile and sustainable alternative for next-generation sensing technologies.

### Chemical sensors based on biomass-derived CQDs

Biomass-derived CQDs have emerged as promising fluorescent nanomaterials for sensing exploitations owing to their low-cost, eco-friendly fabrication routes and tunable optical properties. Among the various applications, chemical sensing—particularly for toxic metal ions and environmentally relevant anions—has received substantial attention. This section explores the potential of biomass-based CQDs in detecting Fe³⁺, Hg²⁺, and Pb²⁺ ions, as well as anions like Cl⁻ and NO₃⁻, discussing their sensitivity, selectivity, and detection mechanisms based on numerous studies conducted between 2021 and 2025 (Table 2).

#### Ferric ion (Fe³⁺)

**Fig. 2:**
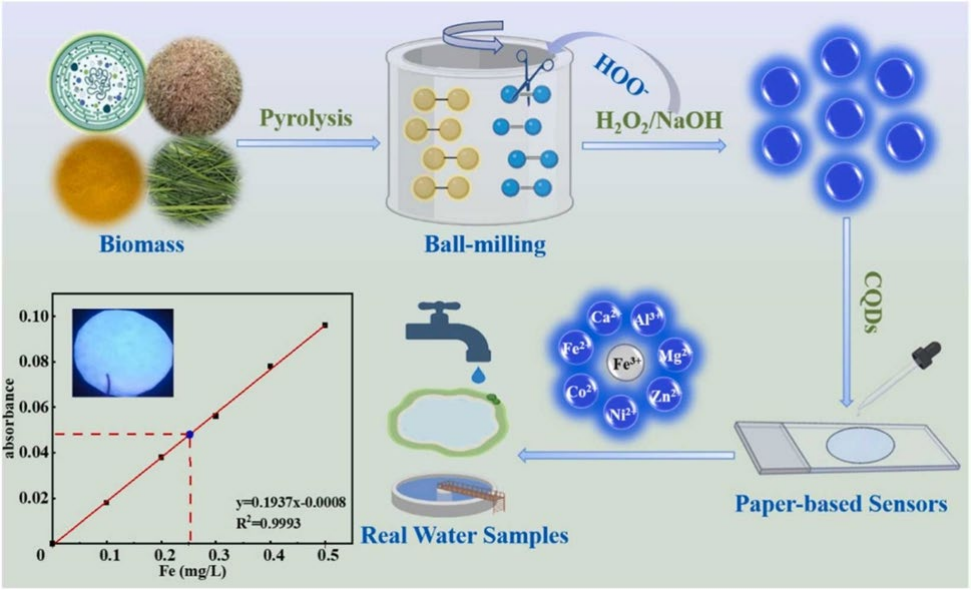
Paper-based sensors from biomass wastes for Fe^3+^ detection. Reproduced from Jagannathan et al. (2021) with permission from Elsevier

Fe³⁺ is an essential trace element, but its excessive accumulation can lead to oxidative stress and organ dysfunction. Numerous biomass-derived CQDs have shown excellent Fe³⁺ sensing capabilities. For instance, CQDs derived from blue-green algae, straw, corn protein meal, and Typha angustifolia demonstrated high sensitivity for Fe³⁺ over a linear span of 0–60 µM with a strong correlation coefficient (R² = 0.9956). These CQDs were integrated into paper-based devices for real-time, field-deployable water testing using a fluorescence quenching mechanism (Jagannathan et al. 2021) (Fig. 2). Similarly, crop biomass containing cellulose and lignin yielded CQDs with dual-band fluorescence from π→π and n→π transitions, providing a broad detection range of 0–500 µM for Fe³⁺ with high selectivity (Raja et al. 2024). Camphor tree leaf-derived CQDs acted as efficient fluorescent probes, achieving a good detection limit of 2.03 µM for Fe³⁺ (Qureashi et al. 2021). In a more multifunctional approach, CQDs synthesized from corn cobs enabled the detection of several analytes including Fe³⁺, with a detection span of 0.310–1.55 µM and an LOD of 1.30 µM (Korram et al. 2023). Notably, these CQDs also detected DNA, paracetamol, and other heavy metals, showcasing their versatility. Macaúba palm fibers, an abundant biomass source, yielded CQDs capable of detecting Fe³⁺ (LOD: 0.69 µM), Cu²⁺, and Hg²⁺ through a fluorescence “turn-off” response. The selectivity was further enhanced by a “turn-on” effect in the existence of ascorbic acid, which reduced Fe³⁺, recovering the fluorescence signal by ~ 70% (Singh et al. 2023). Lotus stem-derived CQDs displayed high aqueous stability and sensitivity, showing a distinctive ON–OFF–ON fluorescence behavior for Fe³⁺ measurement with an LOD of 3.8 µM. This behavior was validated in real water samples, confirming its environmental applicability (George et al. 2023). Banana petiole CQDs offered exceptional sensitivity, with an LOD of 0.21 nM and a linear span of 5–200 nM for Fe³⁺ detection. A paper-based assay showed comparable sensitivity, suggesting its utility for precision agriculture (Zhu et al. 2021). Other high-performance examples include tea leaf CQDs (LOD: 0.079 µM) (Kundu et al. 2022), papaya seed-derived CQDs (LOD: 2.35 µM) (Liu et al. 2023), and lignin-based CQDs (LOD: 0.77 µM) (Mansi and Gaurav 2023).

A critical examination of the reported Fe³⁺ detection limits reveals substantial variability across biomass precursors, even among those with similar lignocellulosic origins. For instance, lignin-derived CQDs exhibit Fe³⁺ LODs ranging from 0.45 to 0.77 µM (Lv et al. 2023, Mansi and Gaurav 2023) to as low as 0.15 µM for rice husk-derived CQDs (He et al. 2023), i.e. a difference of nearly an order of magnitude. This disparity may be partly attributable to several interrelated factors. First, heteroatom doping appears to play a decisive role: nitrogen-doped lignin CQDs demonstrate enhanced electron density and coordination sites for Fe³⁺, lowering detection limits compared to undoped counterparts (Lv et al. 2023). Second, surface functional group speciation likely varies with precursor composition and synthesis conditions; rice husk-derived CQDs possess abundant silanol groups alongside carboxyl and hydroxyl moieties, potentially creating additional Fe³⁺ binding sites that intensify quenching efficiency (He et al. 2023). However, direct causal relationships have not been systematically validated, and differences in measurement conditions (pH, ionic strength, instrument settings) may also contribute significantly. Third, measurement conditions, including buffer pH, ionic strength, and excitation wavelength, are rarely standardized across studies and can profoundly influence apparent sensitivity. For example, Fe³⁺ coordination is highly pH-dependent, and reported LODs optimized at specific pH values may not reflect performance under environmentally relevant conditions. Finally, instrumental factors such as fluorometer slit width, integration time, and detector sensitivity contribute to inter-laboratory variability. Such observations reveal that direct LOD comparisons without consideration of synthesis parameters, surface chemistry, and measurement protocols can be misleading. Future studies should adopt standardized reporting practices that include detailed characterization of surface functional groups (e.g., XPS quantification), doping levels, and measurement conditions to enable meaningful cross-study benchmarking.

#### Mercury ion (Hg²⁺)

 Mercury is a neurotoxic heavy metal of major concern. CQDs derived from pomelo peels exhibited outstanding performance in detecting Hg²⁺ with an ultralow LOD of 0.23 nM in linear span of 0.1–100 µM (Kundu et al. 2022). Strawberry juice-based CQDs also detected Hg²⁺ (LOD: 3 nM) within the same span. A unique approach using chicken feathers allowed for a detection limit of approximately 6.2 nM (Lv et al. 2022), demonstrating that protein-rich biomass sources can also yield functional CQDs. Sugarcane waste-derived CQDs achieved an Hg²⁺ LOD of 0.1 µM (Kundu et al. 2022). The fluorescence quenching in these systems is consistent with strong electron affinity and coordination between Hg²⁺ and oxygen/nitrogen-rich surface groups, although direct evidence (e.g., from X-ray absorption spectroscopy or controlled coordination studies) remains limited in most reports.

#### Lead ion (Pb²⁺)

Pb²⁺ detection has been successfully demonstrated using CQDs synthesized from Platanus acerifolia leaves. These CQDs revealed significant sensitivity with a detection limit as good as 0.02 nM in presence of Cd²⁺, and maintained high selectivity across different multimetal environments. The detection mechanism involves electrochemical sensing via a CQDs/ZIF-8 composite that exploits high surface area and oxygen-containing functional moieties to enhance adsorption and signal response (Abbas et al. 2023). Tulsi leaf-based CQDs also achieved an impressive LOD of 0.59 nM for Pb²⁺ within a 0.010–1.0 µM range (Kundu et al. 2022).

#### Anion detection

 In contrast to metal ion sensing, the detection of anions using biomass-derived CQDs remains in its infancy, with only a limited number of reports available. To date, fluoride ion (F⁻) detection has been demonstrated using CQDs derived from wheat straw (LOD: 49 µM) and taro peel (LOD: 26 µM), while sulfide ion (S²⁻) detection has been achieved with carrot-derived CQDs (LOD: 0.060 µM) (Kundu et al. 2022). Notably, despite being listed among target analytes in some studies, robust detection systems for chloride (Cl⁻) and nitrate (NO₃⁻) using biomass-derived CQDs have yet to be established, representing a significant gap in the literature. The surface functional groups (e.g., amine, hydroxyl, carboxyl) that render CQDs sensitive to metal ions could theoretically be exploited for anion recognition through electrostatic interactions or hydrogen bonding; however, systematic investigations remain scarce. This underdevelopment highlights a critical opportunity for future research to expand the anion sensing repertoire of biomass-derived CQDs, particularly for environmentally relevant species such as nitrates and chlorides in water quality monitoring.

The diverse range of biomass-derived CQDs demonstrates exceptional promise in the development of eco-friendly, low-cost chemical sensors for detecting environmentally and biologically significant ions. With detection limits for Fe³⁺ reaching as low as 0.21 nM (Singh et al. 2023), Hg²⁺ at 0.23 nM (Kundu et al. 2022), and Pb²⁺ at 0.02 nM (Abbas et al. 2023), these sensors offer performance comparable to or exceeding conventional materials. Moreover, their successful application in water, agricultural, and environmental matrices supports their real-world utility. Future research should aim to standardize synthesis protocols, explore underutilized biomass sources, and expand sensing to a broader array of anions including Cl⁻ and NO₃⁻ through advanced functionalization techniques.

**Table 2:** Main details of biomass-derived CQDs in chemical sensors for metal ion detection: and anion detection

Carbon source	Main result	Ref.
Algae, straw, corn protein meal, and Typha angustifolia	Fe³⁺ ions. Linear Range: 0–60 µM. Fluorescence quenching.	(Jagannathan et al. 2021)
Crop biomass are biopolymers, like cellulose, hemicellulose and lignin	Fe^3+^. 0–500 µM.	(Raja et al. 2024)
camphor tree leaves biomass	Fe^3+^. LOD is 2.0 µM.	(Qureashi et al. 2021)
Corn cob	LOD for Pb^2+^, Cu^2+^, Fe^3+^, and Cr^3+^ was1.2 nM, 1.3 nM, 0.86 nM and 2.8 nM, respectively.	(Korram et al. 2023)
Macaúba (Acrocomia aculeate) fibers	LOD: Fe³⁺: 0.69 µM; Cu²⁺: 0.99 µM; Hg²⁺: 0.25 µM.	(Singh et al. 2023)
Lotus stem	Fe³⁺, LOD of 3.8 µM.	(George et al. 2023)
Platanus acerifolia leaves	Lead ions (Pb²⁺); Linear Range: 1 nM–1 µM (Pb²⁺ alone). 50 nM–1 µM (Pb²⁺ with Cd²⁺ or with Cd²⁺ and Cu²⁺). 50 nM–750 nM (Pb²⁺ with Cu²⁺). LOD: 0.04 nM (Pb²⁺ alone). 0.02 nM (Pb²⁺ + Cd²⁺). 0.07 nM (Pb²⁺ + Cu²⁺). 0.04 nM (Pb²⁺ + Cd²⁺ + Cu²⁺)	(Abbas et al. 2023)
Longan peel	Cr^6+^. Linear range of 20–200 µM and a detection limit of 1.4 µM.	(Baragau et al. 2021)
Biomass waste	Borassus flabellifer (Fe³⁺): Range 0.1–400 µM; LOD 2.01 µM; Tea leaves (Fe³⁺): Range 0.1–400 µM; LOD 0.079 µM; Tulsi leaves (Pb²⁺): Range 0.01–1.0 µM; LOD 0.59 nM; Pomelo peels (Hg²⁺): Range 0.1–100 µM; LOD 0.23 nM; Strawberry juice (Hg²⁺): Range 0.1–100 µM; LOD 3 nM; Lemon peel (Cr⁶⁺): Range 0.1–50 µM; LOD 73 nM; Wheat straw (F⁻): Range 1–100 µM; LOD 49 µM; Taro peel (F⁻): Range 1–100 µM; LOD 26.2 µM; Carrots (S²⁻): Range 1–100 µM; LOD 0.06 µM; Eggshell membrane (Hg²⁺): Range 0.1–10 µM; LOD 2.6 µM.	(Kundu et al. 2022)
Banana petioles	Fe^3+^: LOD of 0.21 nM; Linear range from 5.0 to 200 nM.	(Zhu et al. 2021)
Spent tea leaves	Fe^3+^: LOD 0.5 µM.	(Ma et al. 2023)
Glucose	Cr^6+^: LOD of 35 µM. Fe^2+^: LOD of 110 µM	(Kasinathan et al. 2022)
Mopan persimmons	Fe^3+^: LOD of 0.324 µM.	(Zhou et al. 2021)
Sugarcane waste	Hg^2+^: LOD of 0.1 µM	(Eskalen et al. 2024)
Quinoa Saponin	Co^2+^: 20 to 150 µM and LOD of 0.49 µM.	(Šafranko et al. 2021)
Pomegranate peel	Fe^3+^: LOD of 7.49 µM.	(Abbas et al. 2022)
Citrus clementina peel	Fe^3+^: LOD of 4.57 ± 0.27 µM. Span: 7.0 µM − 50.0 µM.	(Zhong et al. 2025)
spent tea leaves	Fe^3+^: LOD of 0.29 ± 0.4 µM.	(Ren et al. 2023)
Peperomia tetraphylla	Fe^3+^: LOD of 2.7 µM.	(Xia et al. 2022)
Agroforestry waste of apple leaves	Fe^3+^: LOD of 4.0 µM. Range 0–160 µM.	(Jose et al. 2024)
Lignin	Fe^3+^: LOD of 0.77 µM. Range 0 ~ 300 µM.	(Mansi and Gaurav 2023)
Flowers of wintersweet	Cr(VI): LOD of 0.07 µM. Range 0.1 to 60 µM. Fe^3+^: LOD of 0.15 µM. Range 0.05 to 100 µM.	(Ren et al. 2024)
Wrightia coccinea	Fe^3+^. LOD is 0.511 µM	(Wang et al. 2023)
Lignin	Fe^3+^ and Co^2+^ ranging from 0.27 to 250 µM and LOD is 0.45–500 µM, respectively.	(Lv et al. 2023)
Lignin	Cu^2+^ in an aqueous system: 2.4 µM.	(Kamarol Zaman et al. 2021)
Spinach	Cr(VI): linear span of 0–220 µM and detection limit (LOD) of 0.24 µM.	(Santiago et al. 2025)
Fruit bunch biochar	Cu^2+^: linear detection range of 0–400 µM and a detection limit of 0.42 µM.	(Yang et al. 2022)
Beetroot waste (bagasse)	Cu^2^⁺.	(Tony Elizabeth et al. 2024)
Corn straw	Cu^2^⁺. LOD of 68 µM. Range: 16 µM to 7.9 mM.	(Keerthana et al. 2023)
Morinda coreia	Fe^3+^. LOD is 1.3 µM	(Zhu et al. 2022)
Water amaranth leaves	Cd^2+^: LOD of 15 nM. Range 0–70 µM.	(Mu et al. 2024)
Lignin	Cr^6+^: LOD of 0.077 µM.	(Bayazeed Alam et al. 2022)
Corn straw	Fe^3+^: LOD of 5.32 µM. Range 50–2000 µM.	(Yin et al. 2024)
Papaya seeds	Fe^3+^: LOD of 2.35 µM.	(Liu et al. 2023)
Chlorophyll	Hg^+^: LOD of 67 pM. Range 100 pM–20 µM. As^3+^: LOD of 1.53 µM. Range 10 − 30 µM.	(Yan et al. 2021)
Saponin	Fe^3+^: LOD of 0.115 µM.	(Preethi et al. 2022)
Bamboo Stems	Fe^3+^: LOD of 0.486 µM. Range 0.01–10 µM.	(Architha et al. 2021)
Rice stock	Cu^2+^: LOD of 0.032 µM. Range 0–260 µM.	(Mohamed et al. 2023)
Chicken feather	Hg^2+^: LOD of 6.2 nM.	(Lv et al. 2022)
Mexican Mint	Fe^3+^: LOD of 53 µM. Range 0–15 µM.	(Tao et al. 2022)
Milk powder	Cu^2+^: LOD of 0.003 µM. Range 0.01–15 µM. Risedronate sodium: LOD of 1.48 µM. Range 5.02–883 µM.	(Raju et al. 2024)

### Biological sensors based on biomass-derived CQDs

Biomass-derived CQDs are increasingly applied in biological sensing exploitations due to the tunable fluorescence, high water solubility, minimal cytotoxicity, and abundant functional groups that interact with biomolecules. Among these, glucose monitoring and pathogen detection stand out as essential applications in medical diagnostics and public health. These CQDs provide versatile platforms for selective and sensitive determination of biological analytes through fluorescence-based mechanisms such as FRET (Förster Resonance Energy Transfer), IFE (inner filter effect), static quenching, and surface charge modulation (Table 3).

#### Glucose monitoring and physiological pH sensing

While glucose-specific detection using biomass CQDs remains less explored than metallic ions, significant advancements have been made in the area of monitoring physiological parameters, such as pH in sweat or related hydrogen peroxide (H₂O₂)-mediated glucose oxidation systems. One prominent example is the use of natural lignocellulose-derived CQDs, which were incorporated into a polyvinyl alcohol (PVA) matrix to create a stable and biocompatible film. These CQDs showed strong fluorescence modulation within the physiological pH range of 4.0 to 7.0, corresponding to typical human sweat pH. The mechanism involved protonation/deprotonation of surface groups, causing observable fluorescence shifts under UV light. This enabled real-time pH monitoring during physical activity, indicating potential for wearable biosensor applications (Padmapriya et al. 2023).

In another case, Moringa oleifera leaf-derived CQDs were employed to detect hydrogen peroxide (H₂O₂), a by-product of enzymatic glucose oxidation. The sensor operated through a FRET-based photoluminescence mechanism, where the presence of H₂O₂ turned the fluorescence “on” or “off” depending on the interaction with surface functionalities. Though the LOD for H₂O₂ was relatively high (26.4 mM), the sensor also effectively detected aspartic acid with a much lower LOD (134 nM), showcasing versatility in detecting multiple bioanalytes in aqueous environments (Sangubotla et al. 2023).

*Neurotransmitter and drug detection*: Dopamine and Related Analytes: Biomass CQDs have also found promising roles in monitoring neurotransmitters such as dopamine (DA), which is crucial for diagnosing neurological disorders. CQDs synthesized from banana flower bracts (Musa acuminata) exhibited remarkable specificity and sensitivity toward dopamine. These CQDs demonstrated an ample linear measurement span of 6.0 µM to 0.1 mM (LOD: ~500 pM), and an even broader span of 2.5 µM to 0.16 mM in real-time dopamine injections (LOD: 630 pM). Notably, the sensor showed excellent anti-interference properties, remaining unaffected by common interferents like uric acid and ascorbic acid (Jia et al. 2023). Similarly, coffee-derived CQDs displayed high fluorescence sensitivity for dopamine detection with an LOD of 4.25 nM in the 0–30 µM span (Qi et al. 2019). These sensors are proposed to operate via π–π interactions or hydrogen bonding between dopamine molecules and CQD surface groups. Static quenching has been suggested as the dominant mechanism in studies that reported unchanged fluorescence lifetimes upon dopamine addition; where such measurements were not performed, the assignment remains tentative.

#### Pathogen and antibiotic detection

In the context of pathogen detection, several studies have utilized CQDs for the indirect identification of bacterial infections via antibiotic sensing. For example, CQDs derived from pitaya peel were used for the fluorescence-based detection of tetracycline antibiotics—tetracycline (TC), oxytetracycline (OTC), and chlortetracycline (CTC). The detection limits were 33.8 nM for TC, 40.5 nM for OTC, and 41.9 nM for CTC in a phosphate-buffered saline system (pH 7.0), with linear ranges extending up to 100 µM (Pang et al. 2022). These CQDs utilized mechanisms such as inner filter effect (IFE), static quenching, and aggregation-induced emission (AIE), enabling high specificity and distinguishability among structurally similar antibiotics. Rice residue-derived N-CQDs also enabled the detection of tetracycline analogues, with LODs of 0.237 µM (tetracycline), 0.374 µM (terramycin), and 0.279 µM (chlortetracycline), demonstrating practical application in antibiotic monitoring (Qandeel et al. 2023). Moreover, CQDs synthesized from onion and cabbage juices were applied to detect nitazoxanide (NTZ), an antiparasitic drug repurposed for viral infections including COVID-19. This sensor achieved an LOD of 0.07 µM over a range of 0.25–50.0 µM. It also demonstrated sensitivity for hemoglobin (Hb), with an LOD of 10.3 nM, making it suitable for use in medical diagnostics and blood analysis (Manjubaashini et al. 2024).

Bird’s nest-derived CQDs successfully detected Vitamin B12 in serum samples with an LOD of 0.24 µM, showing high recovery rates (96%–100%), thus highlighting the potential for clinical biomarker monitoring (Nagaraj et al. 2022).

#### Biocompatibility and cytotoxicity for In Vitro applications

Biological applications require that CQDs exhibit minimal cytotoxicity and high biocompatibility. Several biomass-derived CQDs have demonstrated excellent performance in these aspects. For instance, groundnut and pistachio shell-derived CQDs maintained 90% cell viability and showed hemocompatibility levels as low as 0.1%, even at higher concentrations (John et al. 2023). Likewise, banana stem CQDs and Plumbago indica leaf CQDs reported over 96% cell viability for muscle and kidney cell lines (Goswami et al. 2024, Reagen et al. 2021). Further, graphene quantum dots (GQDs) from cis-cyclobutane-1,2-dicarboxylic acid were successfully internalized into MCF-7 breast cancer cells without inducing toxicity at levels up to 200 µg/mL, making them effective for fluorescence imaging and biosensing (Wu et al. 2023).

**Fig. 3:**
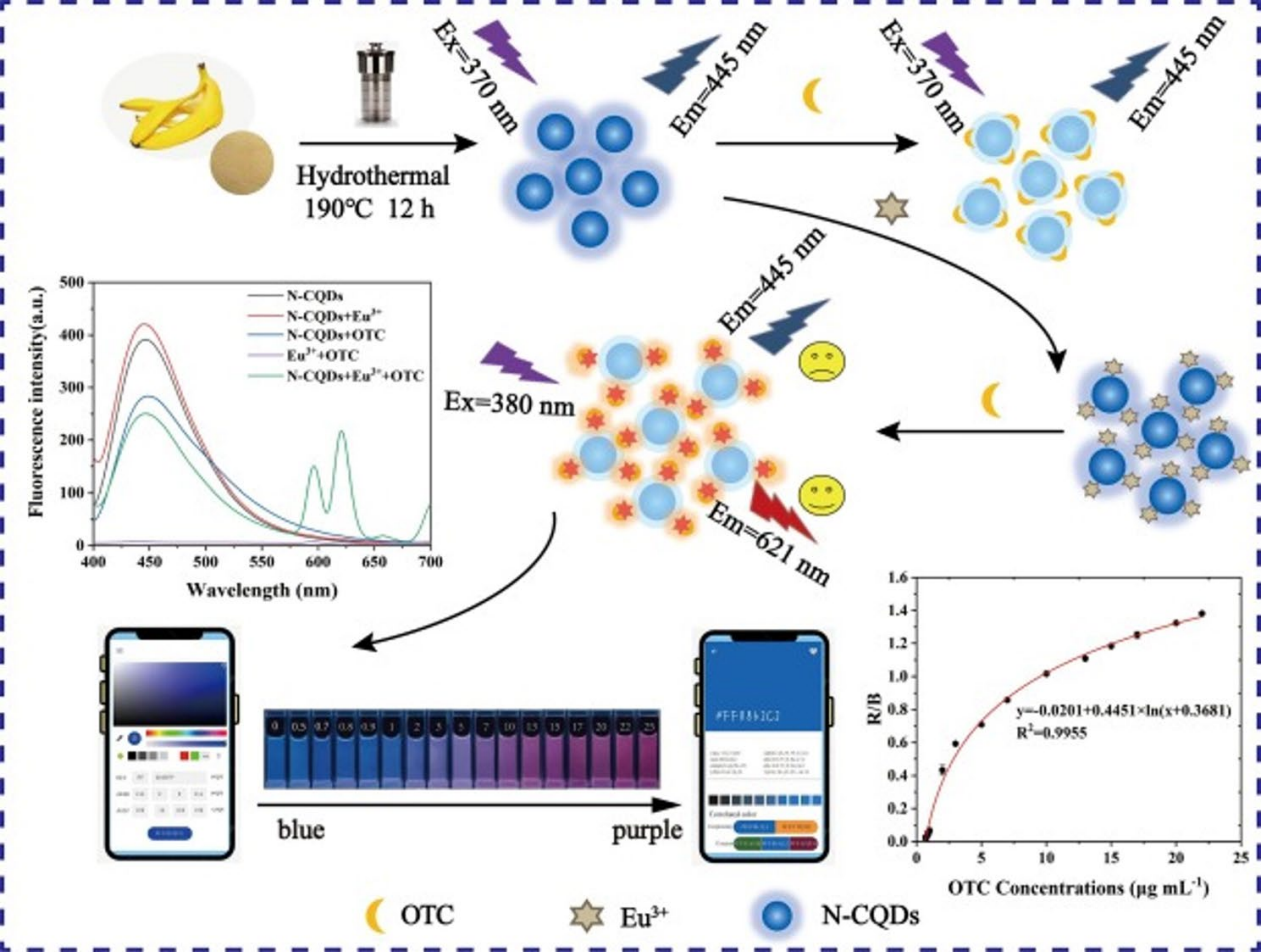
Schematic illustration of OTC sensitivity and visually aided detection using the N-CQDs/Eu³⁺ ratiometric fluorescence sensor. Reproduced from John et al. (2024) with permission from Elsevier

A study performed by Wu et al. (John et al. 2024) presents a ratiometric fluorescence sensor based upon N-doped carbon quantum dots (N-CQDs) from banana peel and europium ions (Eu³⁺) for sensitive and smartphone-assisted visual detection of OTC (oxytetracycline) (Fig. 3). The sensor operates via dual mechanisms: inner filter effect (IFE) quenching N-CQD fluorescence (445 nm) and antenna effect enhancing Eu³⁺ emission (621 nm), enabling a color shift from blue to purple under UV light. Having a low detection limit (0.020 µM) and high recovery rates (96.9–103%) in pork and swine urine, the system offers a portable, cost-effective alternative to traditional methods like HPLC. Smartphone-based RGB analysis further allows on-site, semi-quantitative detection, demonstrating strong potential for food safety and environmental monitoring. Future work could optimize specificity and long-term stability for broader applications.

Biomass-derived CQDs provide a versatile and green platform for developing biological sensors able of detecting a vast spectrum of analytes ranging from glucose intermediates (e.g., H₂O₂) to neurotransmitters and antibiotics. These sensors exhibit low limits of detection (as low as 500 pM for dopamine (Jia et al. 2023) and 33.8 nM for tetracycline (Pang et al. 2022), broad linear ranges, high selectivity, and low cytotoxicity. Their performance in physiological and clinical matrices highlights the potential for real-time, point-of-care diagnostics and wearable biosensors. Continued advancements in surface engineering and nanocomposite integration are expected to expand their capabilities for early disease detection and personalized health monitoring.

**Table 3:** Main details of biomass-derived CQDs in biological sensors

Carbon source	Main result	Ref.
Natural lignocellulose	pH variation in human sweat. Linear range: (approx. 4.0–7.0).	(Padmapriya et al. 2023)
Moringa oleifera (drumstick leaves)	H_2_O_2_ LOD is 26.4 mM; Aspartic acid, LOD is 134 nM.	(Sangubotla et al. 2023)
Rhizomes of Acorus calamus	Rhodamine B (Rh B) and sunset yellow (SY): 0–2 µM; LOD is 96 nM.	(Kaur et al. 2024)
Banana flower bract (Musa acuminata)	Dopamine, LOD is 500 pM and a linear range of 6.0 µM to 0.10 mM.	(Jia et al. 2023)
Rice residue	Fe^3+^; range, 3.32–32.3 µM, with LOD of 0.746 µM. LOD of tetracycline, terramycin and chlortetracycline were 0.237, 0.374 and 0.279 µM, respectively.	(Qandeel et al. 2023)
Groundnut and pista shell	In vitro assays indicated good cell compatibility at 90% and hemocompatibility at 0.3% and 0.1% for GNSE and PSE CQDs, respectively.	(John et al. 2023)
Plumbago indica leaves	Picric acid (PA) with LOD of 18 nM.	(Goswami et al. 2024)
Indian gooseberry	Vitamin B2. LOD) ∼35 nM	(Kaur et al. 2024)
Banana stems	P-CQDs were used to test the cytotoxicity of kidney cells (HEK293) and muscle cells (L6) in vitro. At a concentration of 400 µg/mL, the P-CQD’s cell viability reached its maximum of around 100%, while its lowest was 96.2%.	(Reagen et al. 2021)
Loblolly pine	Temperature sensing, 0–60 °C;	(Shi et al. 2024)
Cis-cyclobutane-1,2-dicarboxylic acid	It was used for cytotoxicity studies using the mouse macrophage cell line RAW 247 and for cell imaging using the human breast cancer cell line MCF-7.	(Wu et al. 2023)
Rice husk	Fe^3+^: LOD is 0.149 µM with an ample linear span of 0–1.30 µM. It demonstrated strong sensitivity and selectivity for both ciprofloxacin (CPX) and ofloxacin (OFX). For OFX and CPX, the computed detection limits were 0.150 µM and 0.127 µM, respectively, with a linearity span of 0.05–1.15 µM.	(He et al. 2023)
Pitaya peel	Tetracycline (TC); 0.2–56.0 µM, Oxytetracycline (OTC); 0.4–100 µM, Chlortetracycline (CTC); 0.4–100 µM. LOD: TC is 33.8; OTC is 40.5; CTC is 41 (PBS buffer)	(Qandeel et al. 2023)
Bird’s nests	Vit B12. The linear span was 0 ∼ 100 µM, LOD was 0.24 µM.	(Nagaraj et al. 2022)
Silk	Salicylic acid, the range of 0.5 to 200 µM with the LOD of 0.14 µM.	(Sun et al. 2023)
Longan Nucleus	Drug Rifampicin. The line span of 0.10–9.0 µM, with a detection limit of 0.96 µM.	(Zhu et al. 2023)
Coffee grounds	Colorimetric and fluorometric procedures for ascorbic acid assay with low LOD (1.56 µM and 0.133 µM, respectively).	(Yin et al. 2021)
Cellulolytic enzyme lignin	Cytochrome c and trypsin: A linear range was 1–50 µM and 0.09–5.4 µM. The LOD valueswere 0.29 µM for Cyt c and 0.013 µM for trypsin.	(Durrani et al. 2023)
Green bean	Fe^3+^: linear range of 10–70 µM and LOD is 3.6 nM. ATP: Range is 50–600 µM LOD is 60 nM.	(Zhu et al. 2021)
Plum	Doxorubicin in human urine and human serum samples: Range is 1–30 µM with LOD of 0.12 µM.	(Li et al. 2021)
Coffee	Dopamine. LOD of 4.2 nM.	(Qi et al. 2019)
Grape seed	The ascorbic acid and Cu(II) assays had LODs 0.3 µM and 0.6 µM, respectively.	(He and Du 2023)
Onion and cabbage juices	Nitazoxanide (NTZ; a drug for COVID-19), in the span of 0.25–50.0 µM with LOD of 0.07 µM. Hemoglobin (Hb) over the range of 36–908 nM with LOD of 10 nM.	(Manjubaashini et al. 2024)
crayfish shell	Tartrazine in food: LOD of 0.48 µM in the linear span of 0–70 µM.	(Li et al. 2023)
Beer	Fe^3+^: Range is 1.0–20 µM and 100–300 µM, respectively. Ascorbic acid: Range is 1 to 200 µM with LOD of 0.84 µM.	(Zhao et al. 2023)
Orange peel	The FA in the span of 5.0–90.00 µM and 0.30–2.5 mM, with LOD of 0.15 µM.	(Wei et al. 2023)
Chitosan	Photo responsiveness (ΔR/R0 ≈ 20%) and pH responsiveness (pH range ≈ 4–7) performance.	(Wang et al. 2023)
Banana peel	Oxytetracycline (OTC): spanning from 0.022–11 µM and 11–54 µM, with LOD of 0.020 µM for OTC.	(John et al. 2024)

### Environmental sensors based on biomass-derived CQDs

Biomass-derived CQDs have gained significant traction in environmental monitoring owing to their low toxicity, fluorescence responsiveness, and environmentally benign fabrication. Their surface-functionalized nanostructures allow them to selectively interact with specific chemical pollutants and gases, making them excellent candidates for sensitive, selective, and portable environmental sensors (Table 4). The primary targets in this context include pesticides, organic pollutants, and gaseous species like ammonia (NH_3_) and formaldehyde (CH_2_O).

#### Pollutant detection

**Fig. 4:**
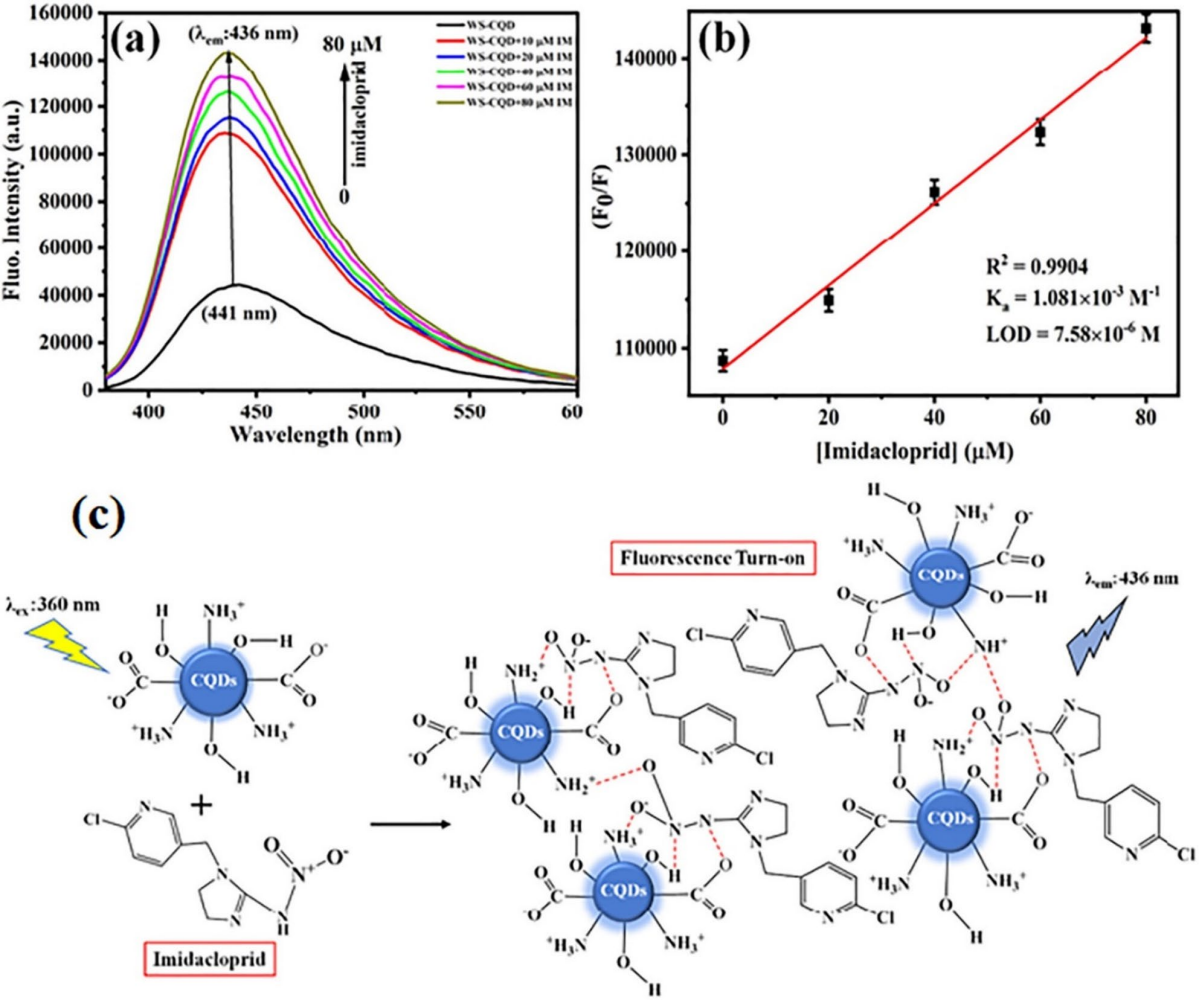
**a** The fluorescence response of WS-CQDs to progressively higher levels of imidacloprid (0–80 µM) when excited at 360 nm (1 nm slit width), **b** The resulting calibration curve showing linear response, and **c** An illustrative diagram depicting the fluorescence enhancement mechanism through WS-CQDs-imidacloprid binding interactions. Reproduced from Li et al. (2021). with permission from Elsevier

Pesticide detection is one of the most critical applications of biomass-derived CQDs given their environmental persistence and toxicity. A compelling example is the paper-based fluorescence sensor developed for detecting glyphosate, a widely used herbicide. Though the exact biomass precursor was not disclosed, the sensor achieved LOD of 0.075 µM in fluorescence spectrofluorometric assays and 0.15 mM using smartphone-based RGB analysis. Glyphosate detection relied on a fluorescence quenching-recovery mechanism: Fe³⁺ first quenched CQD fluorescence via static quenching, which was reversed upon glyphosate addition due to Fe³⁺ chelation. The sensor was validated in food matrices (juice and flour) with recovery rates between 87.6 and 113% (Devi et al. 2023). In another study, CQDs synthesized from walnut shells were utilized to measure imidacloprid, a neonicotinoid insecticide. The sensor offered a linear span of 0–80 µM and an LOD of 7.58 µM, with good correlation (r² = 0.9904), confirming its potential in agricultural residue analysis (Li et al. 2021). For instance, lignin-rich walnut shell-derived CQDs (Li et al. 2021) exhibit the enhanced graphitic character and π-rich surfaces expected from their aromatic precursor chemistry, facilitating interaction with imidacloprid through π–π stacking and ESIPT mechanisms (Fig. 4). In another study, Aloe leaf-derived CQDs were employed to detect nitenpyram, another insecticide, with a broad span of 0.5–200 µM and an LOD of 0.15 µM. The detection mechanism was based on ratiometric fluorescence sensing (F660/F440), providing high specificity and signal reliability (Wang et al. 2024). Biomass CQDs have also shown significant promise in detecting organic pollutants such as pharmaceuticals, dyes, and industrial effluents. For instance, Solanum nigrum leaf-derived CQDs detected phloroglucinol, a phenolic compound used in pharmaceutical and pyrotechnic industries. The CQDs exhibited an ample linear detection range (36–350 nM) and an LOD of 11 nM, with high recovery rates (98.5–101%) from real effluent samples (Keerthana et al. 2023). CQDs from Eclipta alba leaves enabled the detection of multiple organic pollutants. They achieved remarkable LODs of 23.0 nM for trinitrophenol (TNP), 32.9 nM for ciprofloxacin (CIP), and 14.2 pM for morin, showcasing their multi-target sensing capability and extremely low detection thresholds suitable for trace-level contaminant monitoring (Mathew and Mathew 2023). Artemisia annua L.-based CQDs detected sulfanilamide, an antibiotic, with an LOD of 0.22 µM in 50–1600 µM span (Zhao et al. 2024), while cherry tomato stalk-derived CQDs detected oxytetracycline down to 0.018 µM (Arkin et al. 2023). Orange peel CQDs detecting nitric oxide (NO) via FRET with an LOD of 15 nM (Singh et al. 2023). Ginkgo kernel-derived CQDs detecting nitrite (NO₂⁻) in food with a LOD of 0.15 µM, validated by HPLC (Zhang et al. 2022). Loquat fruit-based CQDs detecting permanganate (MnO₄⁻) via inner filter and static quenching mechanisms with a LOD of 0.06 µM (Wang et al. 2022).

#### Gas sensing

Gas sensing, particularly for toxic and industrially relevant gases, is another burgeoning area for biomass CQD applications. Among the most investigated gases are ammonia (NH₃) and formaldehyde (CH₂O) due to their health hazards and presence in agricultural and industrial emissions. Ziziphus mauritiana shell-derived CQDs showed excellent sensitivity to NH₃, achieving a detection limit of 10 nM in aqueous systems through significant fluorescence quenching. This made them a highly selective sensor for ammonia (Ganesan et al. 2022). In a separate study, CQDs were immobilized into a pure cotton face towel (PCFT) derived from expired milk, creating a flexible, wearable sensor. This platform detected NH₃, H₂O₂, and formaldehyde within 16 s, with theoretical detection limits of 12 µM (NH₃), 1.6 µM (H₂O₂), and 10 µM (CH₂O). Such performance enables real-time, on-body environmental sensing (Wu et al. 2022). CQDs from alkali lignin, a waste lignocellulosic product, demonstrated efficient formaldehyde detection in the span of 0.05 to 2.0 mM, with a LOD of 4.64 µM. The detection mechanism involved Schiff base formation and changes in fluorescence emission (Wang et al. 2021). Purslane leaf-derived CQDs showed selective adsorption of formaldehyde on quartz crystal microbalance (QCM) sensors, achieving a sensitivity of 43 Hz·mg⁻¹·L⁻¹ amongst competing organic analytes at ambient temperature (Amer et al. 2021). Additionally, glucose-derived CQDs coated on paper strips exhibited pH responsiveness and detected NH₃ concentrations from 0.1 to 250 µM, signaled by visible color change, supporting use in low-cost, non-instrumental detection (Khan et al. 2024).

Figure 4 demonstrates the fluorescence “turn-on” detection of imidacloprid using walnut shell-derived carbon quantum dots (WS-CQDs), where incremental addition of the insecticide (0–80 µM) causes a 5 nm blue shift (441→436 nm) and enhanced emission intensity due to ground-state complexation between imidacloprid and surface functional moieties (-COOH, -OH, -NH_2_) of WS-CQDs. The sensor shows a linear response (LOD = 7.58 µM, K_a_ = 1.08 × 10⁻³) attributed to excited-state intramolecular proton transfer (ESIPT) between WS-CQDs and imidacloprid’s -NO_2_/-NH groups, which disrupts self-quenching and boosts fluorescence. While this eco-friendly, waste-derived probe offers a simple pesticide monitoring solution, its moderate sensitivity compared to HPLC and need for specificity validation against similar insecticides present limitations for field applications. The study highlights the potential of biomass-derived nanomaterials for sustainable agrochemical detection, though further optimization is needed for trace-level analysis in complex matrices.

Biomass-derived CQDs offer an eco-friendly and highly effective alternative to traditional chemical sensors for environmental applications. Their application in detecting herbicides like glyphosate (LOD: 0.075 µM) (Devi et al. 2023), pesticides like imidacloprid (LOD: 7.58 µM) (Li et al. 2021), industrial organics like phloroglucinol (LOD: 11 nM) (Keerthana et al. 2023), and gases like ammonia (LOD: 10 nM) (Ganesan et al. 2022), demonstrates their superior sensitivity and practical adaptability. These sensors employ a variety of fluorescence modulation mechanisms—including static and dynamic quenching, FRET, and IFE—to achieve low detection limits and high selectivity. Their successful implementation in food matrices, industrial effluents, and wearable platforms marks a critical advancement toward real-time, low-cost environmental monitoring. Future innovations may focus on integrating these sensors with IoT devices for smart agriculture, pollution tracking, and rapid on-site diagnostics.

**Table 4:** Main details of biomass-derived CQDs in environmental sensors

Carbon source	Main result	Ref.
Biomass precursor	Glyphosate (Herbicide). Linear Range is 0.10–0.60 µM and 0.60–1.6 µM. LOD (fluorescence): 0.075 µM.	(Devi et al. 2023)
Ziziphus Mauritiana	NH_3_: LOD 10 nMol in the aqueous medium.	(Ganesan et al. 2022)
Orange peels	NO: LOD and limit of quantification of 15 nM and 50 nM.	(Singh et al. 2023)
Expired milk into pure cotton face towel (PCFT).	The theoretical LOD of the flexible CQD/PCFT sensor for the H_2_O_2_, NH_3_ and CH_2_O was 1.6, 12.0 and 10.0 µM, respectively.	(Wu et al. 2022)
Solanum Nigrum leaves	Phloroglucinol (PL): The sensor exhibited a wide linear range (36–350 nM) and LOD (11 nM) for PL.	(Keerthana et al. 2023)
Alkali lignin	Formaldehyde, 0.05 to 2.0 mM, with LOD of 4.64 µM (based on 3σ/K).	(Wang et al. 2021)
Walnut shell	Linear range of imidacloprid was 0–80 µM with a detection limit of 7.6 µM.	(Devi et al. 2023)
Ginkgo kernel	NO^2−^, having linear span of 0.50–50 µM, the LOD was 0.15 µM.	(Zhang et al. 2022)
Loquat fruit	MnO^4^−: linear span of 0.2–150 µM, LOD of 0.06 µM.	(Wang et al. 2022)
Purslane leaves	Formaldehyde with a high sensitivity that reaches 42.61 Hz mg^− 1^ L.	(Amer et al. 2021)
Aloe leaf	Nitenpyram levelss (0.5–200 µM) with a method detection limit of 0.15 µM.	(Wang et al. 2024)
Artemisia annua L.	Sulfanilamide: Range of 50–1600 µM with LOD of 0.22 µM.	(Zhao et al. 2024)
Eclipta Alba leaves	23.0 nM for trinitrophenol (TNP) and ciprofloxacin (CIP) for 32.9 nM.	(Mathew and Mathew 2023)
Glucose	Ammonia in the range of 0.1–250 µM.	(Khan et al. 2024)
Cherry tomatoes stalk	Antibiotic oxytetracycline from 0.15 µM to 0.018 µM.	(Arkin et al. 2023)

## Mechanisms of sensing

The ability of biomass-derived CQDs to serve as efficient sensors is thought to arise from their highly tunable photoluminescence, which responds sensitively to environmental changes and interactions with analytes. These fluorescence responses—either quenching or enhancement—are believed to be governed by a combination of photophysical and photochemical processes. The following mechanisms have been invoked in the literature, often supported by indirect evidence such as Stern–Volmer analysis, lifetime measurements where available, and spectral overlap calculations. Grasping such mechanisms is necessary to designing selective and sensitive CQD-based sensors for chemical, biological, and environmental applications.

Fluorescence quenching refers to a reduction in emission intensity of CQDs upon interaction with target molecules. This quenching can occur via static or dynamic pathways:

Static Quenching is inferred when the analyte forms a stable, non-fluorescent complex with the CQD in the ground state, typically evidenced by an unchanged fluorescence lifetime upon quenching (in contrast to dynamic quenching, which reduces lifetime). For example, Fe³⁺ and Hg²⁺ ions readily coordinate with surface functional groups (e.g., –COOH, –OH, –NH_2_) of CQDs to form such complexes. In the glyphosate detection system, Fe³⁺ statically quenched the CQD fluorescence, which was subsequently restored (“turn-on”) when glyphosate chelated Fe³⁺ away from the surface, demonstrating reversibility and high specificity (Jagannathan et al. 2021).

Dynamic Quenching involves collision-induced interactions during the excited state of CQDs. This non-radiative deactivation pathway depends on factors like temperature, diffusion rate, and analyte concentration. For instance, the detection of ammonia (NH_3_) by Ziziphus mauritiana CQDs involved dynamic quenching due to the volatile base’s interactions with fluorescent moieties, leading to a rapid decrease in intensity with a detection limit of 10 nM (Raja et al. 2024).

In some cases, fluorescence enhancement (or “turn-on”) is observed, where the analyte disrupts a quenching interaction or stabilizes emissive states. Banana petiole-based CQDs showed increased emission in the presence of Fe³⁺ under specific pH conditions due to stabilization of surface states conducive to radiative decay (Mansi and Gaurav 2023).

Electron transfer plays a pivotal role in sensing mechanisms, especially when CQDs act as either electron donors or acceptors upon interaction with analytes. In the presence of electron-deficient species (e.g., metal ions or nitroaromatics), CQDs can donate electrons from their excited state, leading to fluorescence quenching. Conversely, analytes that serve as electron donors can inject electrons into CQDs, potentially altering the recombination dynamics and either quenching or enhancing fluorescence. For example, detection of phloroglucinol (PL) using Solanum nigrum CQDs relied on photoinduced electron transfer (PET), where the phenolic analyte influenced the surface electron density, altering the emissive state of CQDs and producing a quantifiable signal drop (Singh et al. 2023). Similarly, detection of ascorbic acid (AA) was reported via CQDs/Fe³⁺ complexes, where AA reduced Fe³⁺ to Fe²⁺, reversing quenching through electron donation and restoring fluorescence (Wang et al. 2023).

FRET (Förster Resonance Energy Transfer) is another central mechanism in CQD-based sensors, especially in biological systems. FRET occurs when an excited donor fluorophore (CQD) transfers energy non-radiatively to a nearby acceptor molecule, provided there is spectral overlap betwixt the CQD emission and acceptor absorption, and the two are within 1–10 nm proximity. Biomass-derived CQDs have been effectively used in FRET-based sensing platforms. For example, CQDs synthesized from Moringa oleifera demonstrated a FRET-based “turn-off/turn-on” photoluminescence response for hydrogen peroxide (H₂O₂) and aspartic acid detection. These analytes altered the spatial configuration or electronic environment of the CQD–acceptor system, modulating energy transfer efficiency and resulting fluorescence output (Raja et al. 2024). In another example, orange peel CQDs formed a FRET pair with a naphthalimide unit for nitric oxide (NO) detection, where the fluorescence emission at 530 nm was modulated by analyte-induced changes in FRET efficiency, achieving a detection limit of 15 nM (Qureashi et al. 2021).

Readers should note that many of the mechanistic interpretations summarized here are based on indirect experimental evidence. For example, static versus dynamic quenching is reliably distinguished only when fluorescence lifetime data are available; in several cited studies, only Stern–Volmer plots were provided. Similarly, FRET claims require demonstration of spectral overlap and distance-dependent energy transfer, which are not always rigorously established. The inner filter effect (IFE) is often invoked when absorption spectra overlap with CQD excitation/emission, but this does not exclude other concurrent mechanisms. Where original studies lacked these validating experiments, the mechanistic assignment should be regarded as a plausible interpretation rather than a definitive conclusion.

Beyond experimental distinction, the suitability of each sensing mechanism for real-world applications warrants critical evaluation. Static quenching, while highly sensitive due to ground-state complex formation, can be susceptible to interference from competing ions that disrupt analyte binding, limiting selectivity in complex environmental or biological matrices. Dynamic quenching, being collision-dependent, is less prone to false positives from non-binding interferents but requires careful temperature control and shows narrower linear ranges. FRET-based sensors offer excellent selectivity through molecular recognition elements but demand precise donor-acceptor distance control (< 10 nm) and spectral overlap, making them sensitive to environmental perturbations that alter conformation. The inner filter effect (IFE), while not a true quenching mechanism, provides exceptional robustness in complex samples because it relies on absorption rather than molecular interactions; IFE-based sensors are less affected by ionic strength, pH fluctuations, or nonspecific binding, as demonstrated in successful applications for permanganate (Wang et al. 2022) and tetracycline (Pang et al. 2022) detection in environmental waters. For field-deployable and wearable sensors requiring long-term stability, mechanisms based on IFE or ratiometric fluorescence (which self-calibrates against environmental variability) are generally preferred over single-emission static quenching systems, which may require frequent recalibration.

To provide a systematic overview of how surface chemistry governs sensing behavior, Table 5 summarizes the correlations between specific surface chemical features of biomass-derived CQDs and their dominant sensing mechanisms, along with key advantages and representative analytes.

The structure–mechanism relationships outlined in Table 5 are further enhanced through deliberate heteroatom doping. The mechanistic benefits of nitrogen, sulfur, and phosphorus doping, frequently cited as beneficial in the literature, can now be understood through their direct influence on the electronic phenomena described above. Nitrogen doping introduces electron-rich defects within the sp²-carbon network, creating n-type doping effects that elevate the Fermi level and introduce mid-gap energy states near the conduction band edge (Zhang et al. 2022, Dong et al. 2024). These states serve as additional emissive traps while also providing favorable orbitals for photoinduced electron transfer (PET) to electron-deficient analytes. Sulfur doping, by contrast, distorts the carbon lattice and creates hole-trapping sites that modulate charge recombination dynamics, prolonging excited-state lifetimes and enhancing FRET efficiency (Lv et al. 2023, Yang et al. 2024). Phosphorus doping introduces P = O and P–O functional groups that alter surface polarity and charge distribution, influencing electrostatic interactions with charged analytes (Jia et al. 2023, Li et al. 2023). These doping-induced modifications, i.e. band gap narrowing, altered work function, and modulated charge transfer kinetics, directly govern the efficiency of quenching, PET, and FRET pathways. For example, N-doped CQDs exhibit enhanced static quenching of Fe³⁺ due to stronger coordination at nitrogen-rich binding sites combined with favorable electron transfer from nitrogen-associated energy levels (Kundu et al. 2022, Wang et al. 2023) Thus, doping is not merely a synthetic additive but a fundamental tool for engineering the electronic landscape of CQDs to achieve targeted sensing performance. The efficiency of these mechanisms is intrinsically linked to structural parameters such as defect density, band alignment, and surface functionality, revealing the importance of controlled synthesis for optimizing sensing performance.

In many systems, multiple mechanisms may occur simultaneously or sequentially, enhancing the sensor’s performance. The detection of permanganate (MnO₄⁻) by loquat fruit-derived CQDs involved both inner filter effect (IFE) and static quenching. MnO₄⁻ absorbs excitation or emission light due to spectral overlap with CQDs, decreasing fluorescence intensity while also interacting electrostatically with CQD surfaces to induce further quenching (Liu et al. 2023). Similarly, in the detection of tetracycline antibiotics using pitaya peel-based CQDs, combined mechanisms of static quenching, inner filter effect, and aggregation-induced emission (AIE) contributed to high selectivity and low detection limits (e.g., 34 nM for tetracycline) (Baragau et al. 2021).

The sensing performance of biomass-derived CQDs is governed by a rich array of mechanisms including fluorescence quenching (static and dynamic), electron transfer, and energy transfer (FRET). Each mechanism offers unique advantages in terms of sensitivity, selectivity, and dynamic range. By tuning the CQDs’ surface chemistry and electronic properties, often through controlled synthesis and post-functionalization, these mechanisms can be exploited or combined to construct high-performance sensors for a wast variety of analytes across chemical, biological, and environmental domains. Understanding and leveraging these mechanisms is key to the rational design of next-generation, biomass-based fluorescent nanosensors.

It is important to note that the assignment of specific sensing mechanisms in the cited studies is supported by complementary experimental evidence. Static quenching, for instance, is typically confirmed by fluorescence lifetime measurements, where static quenching shows constant lifetime while dynamic quenching reduces lifetime, as demonstrated in Fe³⁺ detection studies (Jagannathan et al. 2021, George et al. 2023, Wang et al. 2023). FRET-based sensing is validated through spectral overlap analysis between CQD emission and acceptor absorption, along with distance-dependent efficiency calculations (Sangubotla et al. 2023, Singh et al. 2023). Inner filter effect (IFE) is distinguished from true quenching by comparing absorption spectra of the analyte with excitation/emission wavelengths of CQDs and verifying that lifetime remains unchanged (Pang et al. 2022, Wang et al. 2022). These experimental validations, consistently employed across the reviewed literature, ensure that the mechanistic assignments discussed above are robust and reliable.

Beyond the well-established fluorescence quenching and FRET pathways, the sensing performance of biomass-derived CQDs is also governed by more subtle mechanisms such as aggregation-induced emission (AIE), inner filter effects (IFE), and surface charge modulation. For example, antibiotic detection using pitaya peel-derived CQDs demonstrated an AIE mechanism where analyte-induced aggregation enhanced emission intensity, providing both sensitivity and selectivity in complex matrices. Similarly, nitrate and nitrite sensing has been reported to rely strongly on IFE, where spectral overlap between CQD emission and analyte absorption suppresses fluorescence in a concentration-dependent manner. Electrostatic interactions and protonation/deprotonation of surface groups can also modulate fluorescence under varying pH or ionic environments, enabling real-time monitoring of physiological conditions such as sweat pH or metabolic by-products. These multiple, sometimes overlapping mechanisms highlight the multifunctional nature of CQDs and emphasize the importance of correlating fluorescence responses with structural features and surface states. Understanding such synergistic processes will be essential to rationally design CQDs with tailored mechanisms that rival or exceed the performance of existing sensing platforms. The efficiency of these mechanisms is intrinsically linked to structural parameters such as defect density, band alignment, and surface functionality, revealing the importance of controlled synthesis for optimizing sensing performance.

The substantial variation in reported detection limits for identical analytes across different biomass-derived CQDs, for example, Fe³⁺ LODs ranging from 0.21 nM (banana petiole (Kundu et al. 2022)) to 7.49 µM (pomegranate peel (Abbas et al. 2022)), can now be understood through the mechanistic lens established above. This variability does not indicate inconsistency but rather reflects how the efficiency of the dominant sensing mechanism is governed by specific material properties. CQDs with higher quantum yields, optimized heteroatom doping (e.g., N, S), and abundant surface binding sites (carboxyl, amine groups) exhibit stronger analyte interactions, enabling more efficient static quenching or electron transfer and thus lower LODs. Conversely, CQDs with fewer functional groups, lower graphitization, or suboptimal surface states may rely on weaker dynamic quenching or IFE, resulting in higher LODs. Differences in experimental conditions (pH, buffer systems, excitation wavelengths) further modulate these mechanisms by altering surface charge or spectral overlap. Thus, the observed variability reveals the tunability of biomass-derived CQDs and reinforces the central premise of our structure–mechanism–performance framework (Fig. 1): that rational design, i.e. linking precursor selection and synthesis parameters to targeted mechanisms, is essential for optimizing sensing performance.

The practical selectivity of biomass-derived CQDs is quantitatively expressed through selectivity coefficients (K_sel_ = response to interferent/response to target). Values below 0.1 are generally considered acceptable, and several studies have reported such performance. For example, banana petiole-derived CQDs exhibited Fe³⁺ selectivity coefficients of 0.02–0.05 against Ca²⁺, Mg²⁺, Na⁺, and K⁺, enabling accurate detection in agricultural water samples (Kundu et al. 2022). Similarly, Platanus acerifolia leaf-derived CQDs demonstrated selective Pb²⁺ detection even in the presence of Cd²⁺ and Cu²⁺ (Abbas et al. 2023). Beyond selectivity, matrix tolerance, i.e., the ability to maintain accurate response in complex real-world samples, is critically dependent on the dominant sensing mechanism. The inner filter effect (IFE) offers inherent robustness because it relies on analyte absorption rather than molecular recognition; this has been exploited successfully for permanganate detection in environmental waters (Wang et al. 2022) and tetracycline detection in biological fluids (Pang et al. 2022). In contrast, static quenching and PET-based sensors are more susceptible to interference from non-target matrix components such as humic acid or proteins. Researchers are encouraged to report not only spiked recovery percentages but also matrix effect percentages (recovery in matrix divided by recovery in buffer) and to systematically investigate common interferents, including humic substances, bovine serum albumin, and variable ionic strength (Jagannathan et al. 2021, He et al. 2023, John et al. 2024). These practices will enable more meaningful cross-study comparisons and accelerate the translation of CQD sensors from laboratory demonstrations to real-world applications.

**Table 5:** Correlation between surface chemical features of biomass-derived CQDs and their dominant sensing mechanisms

Surface chemical feature	Preferred sensing mechanism	Key advantages	Typical analytes	Ref.
Carboxyl-rich (–COOH)	Static quenching (coordination)	High sensitivity, selective for metal ions	Fe³⁺, Hg²⁺, Cu²⁺	(Jagannathan et al. 2021, George et al. 2023, Wang et al. 2023)
Amine-rich (–NH₂)	Photoinduced electron transfer (PET)	Fast response, good for electron-deficient analytes	Nitroaromatics, pesticides	(Kundu et al. 2022, Wang et al. 2023)
Hydroxyl-rich (–OH)	Hydrogen bonding + dynamic quenching	Broad applicability, pH-responsive	Dopamine, glucose	(Jia et al. 2023, Qi et al. 2019)
N-doped (graphitic N, pyridinic N)	Enhanced electron transfer, n→π* transitions	Higher quantum yield, lower LODs	Fe³⁺, tetracyclines	(Lv et al. 2023, Kundu et al. 2022, Qandeel et al. 2023)
S-doped (thiophene S)	Prolonged excited-state lifetime, enhanced FRET	Improved FRET efficiency	H₂O₂, aspartic acid	(Lv et al. 2023, Yang et al. 2024, Sangubotla et al. 2023)
P-doped (P= O, P-O)	Altered surface charge, electrostatic interactions	Good for charged analytes	Dopamine, antibiotics	(Jia et al. 2023, Wei et al. 2023)
π-rich aromatic domains	π-π stacking + static quenching	Selective for aromatic pollutants	Pesticides, antibiotics	(Lv et al. 2023, Pang et al. 2022, Mansi and Gaurav 2023)
High surface oxidation	IFE (due to strong absorption)	Robust in complex matrices, less interference	MnO₄⁻, nitrite	(Pang et al. 2022, Wang et al. 2022)

## Benchmarking against conventional analytical methods

Biomass-derived CQDs have achieved impressive detection limits for numerous analytes, often matching or exceeding those of conventional techniques such as ICP-MS, atomic absorption spectroscopy (AAS), and HPLC-MS/MS. Reported Fe³⁺ LODs as low as 0.21 nM (Zhu et al. 2021), Pb²⁺ LODs of 0.020 nM (Abbas et al. 2023), Hg²⁺ LODs of 0.23 nM (Kundu et al. 2022), and tetracycline LODs of 34 nM (Pang et al. 2022) compare favorably with typical LODs of ICP-MS (pM–fM range after digestion (Universidade de Santiago de Compostela 2024 , Dunnivant 2008) and HPLC-MS/MS (nM–pM range after extraction (Dluhošová et al. 2024, Guo et al. 2016)). However, detection limits alone provide an incomplete picture of analytical value. In practice, selectivity, matrix tolerance, robustness, and sample preparation requirements are equally important determinants of whether a sensor can be deployed outside the laboratory.

### Selectivity

Biomass-derived CQDs often achieve high selectivity through rational surface functionalization, heteroatom doping, or molecular recognition elements. As noted in Sect. 6, selectivity coefficients below 0.1 against common interferents are routinely reported (Zhu et al. 2021, Ma et al. 2023). Nevertheless, selectivity against chemically similar competing ions (e.g., Cu²⁺ interference in Fe³⁺ detection, or Cd²⁺ interference in Pb²⁺ detection) is less frequently documented. Conventional techniques like ICP-MS and HPLC-MS/MS achieve excellent selectivity through mass discrimination or chromatographic separation, but at the cost of longer analysis times and more complex instrumentation (Dunnivant 2008, Guo et al. 2016).

### Matrix tolerance

This remains a major challenge for CQD-based sensors. Most validation studies report spiked recovery rates (85–115%) in simple buffers or after extensive dilution, which poorly represent real-world complexity. Environmental matrices contain humic substances and variable ionic strength; biological fluids contain proteins and lipids that can adsorb onto CQD surfaces, quench fluorescence non‑specifically, or alter analyte binding kinetics. For instance, while CQDs have been successfully applied in agricultural water (Zhu et al. 2021, Jagannathan et al. 2021), pork and swine urine (John et al. 2024), and food samples (Pang et al. 2022), systematic investigations of matrix-induced signal suppression relative to buffer controls are scarce. Conventional methods, by contrast, have well-established protocols for matrix-matched calibration, internal standards, and sample cleanup (digestion, solid-phase extraction) that ensure reliable performance even in complex matrices (Universidade , Dluhošová et al. 2024).

### Robustness

Long-term storage stability, operational lifetime, photostability, and batch-to-batch reproducibility are rarely quantified in the CQD literature. While some biomass-derived CQDs retain fluorescence for weeks under refrigeration, inter-batch variability in sensor response (e.g., relative standard deviation of LOD or calibration slope across independent syntheses) is seldom reported. Reference (Jing et al. 2025) critically highlights reproducibility roadblocks and the urgent need for standardization in carbon dot synthesis. Conventional analytical instruments undergo routine calibration, quality control, and inter-laboratory validation, offering documented robustness that meets regulatory requirements (Dunnivant 2008). Future CQD studies should report batch-to-batch reproducibility metrics and adopt accelerated aging protocols to assess long-term stability.

### Sample preparation and cost

These represent clear advantages for CQD-based sensors. Most operate on a “dilute-and-shoot” principle, requiring no digestion, extraction, or derivatization. This simplicity reduces analysis time from hours to minutes and enables field-deployable formats such as paper strips (Jagannathan et al. 2021, Devi et al. 2023) or smartphone-readable devices (Zhu et al. 2021, John et al. 2024). Reagent and consumable costs are typically under USD 1 per test. Conventional methods demand extensive sample preparation, expensive instrumentation, and trained personnel, with per-sample costs often exceeding USD 10–100 (Universidade , Dluhošová et al. 2024).

### Complementary roles

Taken together, biomass-derived CQDs and conventional techniques occupy complementary niches. CQDs excel in simplicity, speed, low cost, and field-deployability, making them ideal for initial screening or point-of-use monitoring where matrix complexity is low. Conventional techniques remain the gold standard for confirmatory analysis, regulatory compliance, and applications requiring high matrix tolerance or trace-level quantitation in complex matrices. Future benchmarking studies should move beyond reporting only LODs and include standardized metrics for selectivity coefficients, matrix effect percentages, inter-batch relative standard deviations, and long-term stability to enable meaningful cross-platform comparisons (Teli et al. 2025, Mathew and Mathew 2023, Jing et al. 2025).

## Challenges and future perspectives

Despite the impressive progress of biomass-derived carbon quantum dots (CQDs) in sensing applications, several interconnected challenges must be addressed before they can transition from laboratory prototypes to practical, field‑deployable, and commercially viable devices. These challenges are organized below into thematic subsections, each followed by corresponding future perspectives.

### Reproducibility and batch‑to‑batch variability

Variability in biomass feedstocks (e.g., seasonal composition changes) and reliance on low‑throughput synthesis methods often lead to inconsistent yields, particle sizes, and fluorescence properties. Few studies report batch‑to‑batch reproducibility of sensor response (e.g., relative standard deviation of detection limits or calibration slopes across three or more independent syntheses) (Jing et al. 2025). Adoption of continuous‑flow reactors, automated platforms, and in‑line monitoring can improve reproducibility. Statistical validation of inter‑batch variability should become a standard reporting requirement.

### Matrix effects and selectivity

Most validations are performed in simple buffers or spiked real samples after dilution, giving spiked recovery rates of 85–115%. However, environmental matrices contain humic acid and variable ionic strength, while biological fluids contain proteins and lipids that can non‑specifically quench fluorescence or adsorb onto CQD surfaces (Pang et al. 2022, Jagannathan et al. 2021, He et al. 2023). Selectivity coefficients against chemically similar interferents (e.g., Cu²⁺ vs. Fe³⁺) are rarely reported (Abbas et al. 2023). Researchers should report matrix effect percentages (recovery in matrix/recovery in buffer) and selectivity coefficients (K_sel_). Surface passivation (e.g., PEGylation, polymer encapsulation) can mitigate non‑specific interference.

### Long-term operational stability

 pH variation, ionic strength changes, and biofouling remain under‑evaluated. While some CQDs retain fluorescence for weeks under refrigeration, systematic assessments across environmentally relevant pH ranges (4–9 for waters, 1–8 for gastric fluids) and high‑salinity media are scarce (Teli et al. 2025, Zhang et al. 2022). Standardized accelerated aging protocols, long‑term monitoring under simulated real‑world conditions, and strategies such as polymer coating or covalent immobilization are needed to establish operational lifetime. *Standardization of synthesis and characterization protocols*: The lack of standardized protocols for quantum yield measurement (reference standards, excitation wavelengths, instrument calibration) and for precursor preparation/purification leads to poor cross‑study comparability (Jing et al. 2025). Adoption of robust metrics (e.g., quantum yield, surface chemistry quantified by XPS) and reporting guidelines that include doping levels, pH, ionic strength, and instrument settings (Ayiloor Rajesh et al. 2022, Zhou et al. 2022), are necessary in the future research.

### Translation to practical devices and regulatory considerations

 Device‑level challenges include: (i) calibration standardization for smartphone‑based readouts (Zhu et al. 2021, John et al. 2024); (ii) signal drift during continuous monitoring (Padmapriya et al. 2023, Wang et al. 2023); (iii) regeneration for reusable applications; (iv) need for internal references or ratiometric designs (Raju et al. 2024, Wu et al. 2023, Arkin et al. 2023). Furthermore, regulatory pathways remain unexplored: biomedical devices require ISO 10,993 compliance; food contact applications must meet EU 1935/2004 or FDA requirements; and CQDs as novel nanomaterials face REACH classification. Engineering solutions (microfluidics, integrated reference channels) and early consultation with regulatory scientists are essential. Long‑term safety data (biodistribution, clearance, chronic toxicity) must be generated in animal models.

### Future trends and new applications

Integration with Internet of Things (IoT) sensors, wearable/implantable devices, and wireless data communication will enable real‑time environmental, agricultural, and clinical monitoring. AI and machine learning can assist in pattern recognition for complex analyte mixtures. Green synthesis innovations (solvent‑free routes, closed‑loop recycling) will further enhance sustainability.

## Conclusion

In recent five years, the CQDs produced from biomass have been a revolutionary new generation of nanomaterials for sensor technology to offer an inexpensive, sustainable, and high-performance alternative to conventional quantum dots. This review has exemplified the diversity of progress accomplished during the timeframe of 2021 to 2025, highlighting the variety of biomass feedstocks like fruit peels, plant leaves, and crop residues in the synthesis of CQDs with highly tunable optical and surface properties. Adoption of pyrolytic, microwave-assisted, and hydrothermal approaches has made efficient and quick synthesis of CQDs possible, with reaction times reduced to a minimum of 10 min in some microwave processes and quantum yields reaching 30% in heteroatom-doped materials.

The sensor attributes of these CQDs have been particularly attractive. Biomass-derived CQDs exhibited remarkable selectivity and sensitivity to a wide variety of analytes, from heavy metal ions (Fe³⁺, Hg²⁺, Pb²⁺) and environmentally relevant anions (NO₃⁻, F⁻), to biomolecules of biological significance (dopamine, antibiotics) and toxic contaminants (glyphosate, NH₃, CH₂O). Notably, CQDs isolated from Solanum nigrum leaves achieved detection limits as low as 8 nM for Fe³⁺, and CQDs from pitaya peel enabled ultrasensitive detection of antibiotics based on aggregation-induced emission effects. The underlying sensing mechanisms as interpreted in the literature (including static/dynamic quenching, electron transfer, and FRET) have been associated with nanomolar or even picomolar detection limits, placing biomass CQDs at the forefront of ultrasensitive and selective sensing platforms.

Despite these advances, some issues persist. Scalability of synthesis, physicochemical reproducibility, and integration of CQDs into stable, real-time sensor devices are still critical bottlenecks. Standardization of fabrication protocols and comprehensive safety profiling will be required for industrial and clinical translation of CQD-based sensors. The field also faces a paradigm shift using machine learning algorithms for pattern recognition for difficult analyte mixtures, ratiometric and multiplexed detection platform innovations, and the use of CQDs for IoT-enabling sensor networks.

The structure–mechanism–performance framework presented herein provides a rational basis for correlating synthesis conditions with sensing efficiency, guiding the predictive design of next-generation biomass-derived CQD sensors. In the future, continuous innovation in green synthesis, functionalization strategies, and device fabrication has the potential to unlock new realms for biomass-derived CQDs. Their inherent environmental friendliness, biocompatibility, and adaptability make them top candidates for next-generation sensor platforms for precision diagnostics, environmental sensing, and smart agriculture. With continued multidisciplinary collaboration and harmonization of rules, biomass-derived CQDs have the potential to power cost-effective and sustainable sensor technologies for a wide range of analytical applications.

## Data Availability

All data generated or analysed during this study are included in this published article.

## References

[CR65] Abbas A et al (2022) Eco-friendly sustainable synthesis of graphene quantum dots from biowaste as a highly selective sensor. Nanomaterials. 10.3390/nano12203696 36296886 10.3390/nano12203696 PMC9609711

[CR58] Abbas A et al (2023) One-step green synthesis of biomass-derived graphene quantum dots as a highly selective optical sensing probe. Mater Today Chem 30:101555

[CR64] Šafranko S et al (2021) Preparation of multifunctional N-doped carbon quantum dots from citrus clementina peel: investigating targeted pharmacological activities and the potential application for Fe3 + sensing. Pharmaceuticals. 10.3390/ph14090857 34577557 10.3390/ph14090857 PMC8465261

[CR1] Ahmed HEH, Soylak M (2024) Exploring the potential of carbon quantum dots (CQDs) as an advanced nanomaterial for effective sensing and extraction of toxic pollutants. TRAC Trends Anal Chem 180:117939–117939

[CR2] Al-Ghamdi SA et al (2023) Biological synthesis of novel carbon quantum dots using Halimeda opuntia green algae with improved optical properties and electrochemical performance for possible energy storage applications. Int J Electrochem Sci 18(5):100102–100102

[CR3] Alhazzani K et al (2023) A reliable ratiometric fluorescence sensing of heparin and its antidote based on cationic carbon quantum dots and acid red 87. Microchem J 190:108666–108666

[CR4] Alomar T, AlMasoud N, Mansour FR (2024) A green method for the preparation of carbon quantum dots from yellow lupin peel waste for spectrofluorometric determination of nirmatrelvir. Spectrochim Acta Part A Mol Biomol Spectrosc 322:124825–124825 10.1016/j.saa.2024.124825 39033610

[CR124] Amer WA et al (2021) Green synthesis of carbon quantum dots from purslane leaves for the detection of formaldehyde using quartz crystal microbalance. Carbon 179:159–171

[CR126] Amin Foisal R, Imran AB, Chowdhury AN (2025) Eco-friendly biomass‐based carbon dots, carbon nanotubes, graphene, and their derivatives for enhanced oil recovery: a new horizon for petroleum industry. ChemistryOpen 14:e202400353 10.1002/open.202400353 PMC12256935 40302426

[CR83] Architha N et al (2021) Microwave-assisted green synthesis of fluorescent carbon quantum dots from Mexican Mint extract for Fe3 + detection and bio-imaging applications. Environ Res 199:111263 33939978 10.1016/j.envres.2021.111263

[CR117] Arkin K et al (2023) Construction of dual-channel ratio sensing platform and molecular logic gate for visual detection of oxytetracycline based on biomass carbon dots prepared from cherry tomatoes stalk. Chem Eng J 464:142552

[CR5] Ayiloor Rajesh G et al (2022) Carbon dots from natural sources for biomedical applications. Part Part Syst Charact 39(9):2200017–2200017

[CR59] Baragau I-A et al (2021) Efficient continuous hydrothermal flow synthesis of carbon quantum dots from a targeted biomass precursor for on–off metal ions nanosensing. ACS Sustain Chem Eng 9(6):2559–2569

[CR79] Bayazeed Alam M et al (2022) Deciphering interaction between chlorophyll functionalized carbon quantum dots with arsenic and mercury toxic metals in water as highly sensitive dual-probe sensor. J Photochem Photobiol A 431:114059

[CR29] Chen J et al (2024) Natural biomass carbon dots-based fluorescence sensor for high precision detection of vitamin B12 in serum. Spectrochim Acta Part A Mol Biomol Spectrosc 305:123459 10.1016/j.saa.2023.123459 37827002

[CR6] Chen W et al (2022) Fluorescent probe of nitrogen-doped carbon dots derived from biomass for the sensing of MnO4 – in polluted water based on inner filter effect. Adv Compos Hybrid Mater 5(3):2378–2386

[CR18] Das GS et al (2024) Nanocarbon-based sensors for the structural health monitoring of smart biocomposites. Nanoscale 16(4):1490–1525 38186362 10.1039/d3nr05522a

[CR112] Devi MS et al (2023) Walnut shell biomass waste derived excitation-dependent CQDs for toxic insecticide sensing and protein denaturation inhibition: an ecofriendly and sustainable approach. Diam Relat Mater 136:110021

[CR28] Dhandapani E et al (2023) A potential forecast of carbon quantum dots (CQDs) as an ultrasensitive and selective fluorescence probe for Hg (II) ions sensing. Mater Sci Engineering: B 287:116098

[CR47] Ding S et al (2021) Green synthesis of biomass-derived carbon quantum dots as fluorescent probe for Fe3 + detection. Inorg Chem Commun 130:108636

[CR130] Dluhošová S et al (2024) Dairy chain safety in the context of antibiotic residues-current status of confirmatory liquid chromatography methods: a review. Antibiotics 13(11):1038 39596733 10.3390/antibiotics13111038 PMC11591054

[CR128] Dong X et al (2024) Probing the fluorescence quenching mechanism of N-doped carbon quantum dots by inorganic ions. Microchem J 197:109854

[CR131] Dunnivant FM (2008) Chap. 5: Comparison of techniques. In: Environmental laboratory exercises for instrumental analysis. Whitman College. http://people.whitman.edu/~dunnivfm/FAASICPMS_Ebook/CH5/CH5.html. Accessed 24 Feb 2026

[CR104] Durrani S et al (2023) Biomass-based carbon dots for Fe3 + and adenosine triphosphate detection in mitochondria. ACS Appl Nano Mater 6(1):76–85

[CR7] Elizabeth AT et al (2023) Green synthesis of value-added nitrogen doped carbon quantum dots from Crescentia cujete fruit waste for selective sensing of Fe3 + ions in aqueous medium. Inorg Chem Commun 149:110427–110427

[CR63] Eskalen H et al (2024) Carbon quantum dots derived from pomegranate peel: highly effective Fe(III) sensor. Biomass Convers Biorefinery 14(1):1201–1214

[CR121] Ganesan S et al (2022) Microwave-assisted green synthesis of multi-functional carbon quantum dots as efficient fluorescence sensor for ultra-trace level monitoring of ammonia in environmental water. Environ Res 206:112589 34929186 10.1016/j.envres.2021.112589

[CR54] George HS et al (2023) Green synthesis of biomass derived carbon dots via microwave-assisted method for selective detection of Fe3 + ions in an aqueous medium. Inorg Chem Commun 157:111348

[CR94] Goswami J et al (2024) Biomass-derived phosphorous-doped carbon quantum dots (P-CQD): an excellent biocompatible material for in-vitro cell imaging. Inorg Chem Commun 162:112276

[CR8] Guan H, Wang D, Sun B (2022) Dual-mode colorimetric/fluorometric sensor for the detection of glutathione based on the peroxidase-like activity of carbon quantum dots. Inorg Chem Commun 136:109147–109147

[CR9] Gulati S et al (2023) Eco-friendly and sustainable pathways to photoluminescent carbon quantum dots (CQDs). Nanomaterials 13(3):554–554 36770515 10.3390/nano13030554 PMC9920802

[CR132] Guo M et al (2016) Determination of tetracycline antibiotics in environmental water using magnetic solid phase extraction combined with high performance liquid chromatography-tandem mass spectrometry. Chin J Chromatogr 34(4):407–413

[CR46] Hao H-C et al (2023) Preparation of high-yield carbon quantum dots and paper-based sensors from biomass wastes by mechano-chemical method. J Environ Chem Eng 11(6):111406

[CR107] He L, Du H (2023) Detection of tartrazine with fluorescence sensor from crayfish shell carbon quantum dots. J Food Compos Anal 118:105200

[CR57] He M et al (2023) Preparation of biomass water-soluble carbon quantum dots and their application in Cr (VI) ions detection. Packaging Technol Sci 36(6):465–472

[CR10] Hu F et al (2024) Zinc-doped carbon quantum dots-based ratiometric fluorescence probe for rapid, specific, and visual determination of tetracycline hydrochloride. Food Chem 431:137097–137097 37572485 10.1016/j.foodchem.2023.137097

[CR49] Jagannathan M et al (2021) Green synthesis of white light emitting carbon quantum dots: Fabrication of white fluorescent film and optical sensor applications. J Hazard Mater 416:125091 33866289 10.1016/j.jhazmat.2021.125091

[CR89] Jia Y et al (2023) Nitrogen doped biomass derived carbon dots as a fluorescence dual-mode sensing platform for detection of tetracyclines in biological and food samples. Food Chem 402:134245 36174349 10.1016/j.foodchem.2022.134245

[CR133] Jing HH et al (2025) Reproducibility roadblocks and standardization in carbon dot synthesis: a critical review of current practices, challenges, and future directions. Microchim Acta 192(12):835 10.1007/s00604-025-07683-z 41269331

[CR93] John BK et al (2023) Bioresource-derived multifunctional carbon quantum dots as a fluorescence and electrochemical sensing platform for picric acid and noncytotoxic food storage application. J Ind Eng Chem 126:546–556

[CR97] John BK et al (2024) Biomass derived carbon quantum dots as a versatile platform for fluorescent sensing, catalytic reduction, fluorescent ink and anticancer agents. Mater Today Sustain 26:100715

[CR69] Jose J et al (2024) Turn-off fluorescence sensor for the detection of ferric ion in water using green synthesized Wrightia coccinea carbon quantum dot. J Fluoresc 35:3343–3353 38767724 10.1007/s10895-024-03760-3

[CR72] Kamarol Zaman AS et al (2021) Properties and molecular structure of carbon quantum dots derived from empty fruit bunch biochar using a facile microwave-assisted method for the detection of Cu2 + ions. Opt Mater 112:110801

[CR61] Kasinathan K et al (2022) Green synthesis of multicolour fluorescence carbon quantum dots from sugarcane waste: investigation of mercury (II) ion sensing, and bio-imaging applications. Appl Surf Sci 601:154266

[CR98] Kaur M, Bhattacharya M, Maity B (2024) Green transformation of biomass-derived Indian gooseberry into fluorescent intrinsic nitrogen-functionalized carbon quantum dots for real-time detection of vitamin B 2 in the nanomolar range. RSC Sustain 2(5):1472–1486

[CR20] Kaushik J et al (2022) Thiourea-functionalized graphene aerogel for the aqueous phase sensing of toxic Pb (II) metal ions and H2O2. Chemosphere 287:132105 34826890 10.1016/j.chemosphere.2021.132105

[CR76] Keerthana P et al (2023) A ratiometric fluorescent sensor based on dual-emissive carbon dot for the selective detection of Cd2+. J Environ Chem Eng 11(2):109325

[CR114] Keerthana P et al (2023) Biomass derived carbon quantum dots embedded PEDOT/CFP electrode for the electrochemical detection of phloroglucinol. Electrochim Acta 448:142184

[CR125] Khan A, Ezati P, Rhim J-W (2024) pH indicator integrated with carbon quantum dots of glucose to monitor the quality of fish and shrimp. Food Bioprocess Technol 17(2):554–569

[CR11] Kolaprath MKA, Benny L, Varghese A (2023) A facile, green synthesis of carbon quantum dots from Polyalthia longifolia and its application for the selective detection of cadmium. Dyes Pigm 210:111048–111048

[CR52] Korram J et al (2023) Biomass-derived carbon dots as nanoprobes for smartphone–paper-based assay of iron and bioimaging application. ACS Omega 8(34):31410–31418 37663469 10.1021/acsomega.3c03969 PMC10468929

[CR12] Kundu A, Basu S, Maity B (2023) Upcycling waste: citrus limon peel-derived carbon quantum dots for sensitive detection of tetracycline in the nanomolar range. ACS omega 8(39):36449–36459 37810728 10.1021/acsomega.3c05424 PMC10552100

[CR55] Kundu A, Maity B, Basu S (2022) Rice husk-derived carbon quantum dots-based dual-mode nanoprobe for selective and sensitive detection of Fe3 + and fluoroquinolones. ACS Biomaterials Sci Eng 8(11):4764–4776 10.1021/acsbiomaterials.2c00798 36200295

[CR13] Li J, Ma X (2024) Preparation of lignin-based full-color carbon quantum dots and their multifunctionalization with waterborne polyurethanes. Int J Biol Macromol 265:130860–130860 38490397 10.1016/j.ijbiomac.2024.130860

[CR106] Li J, Xu O, Zhu X (2021) A facile green and one-pot synthesis of grape seed-derived carbon quantum dots as a fluorescence probe for Cu (ii) and ascorbic acid. RSC Adv 11(54):34107–34116 35497280 10.1039/d1ra05656e PMC9042380

[CR56] Liu Q et al (2023) Sensitive and selective electrochemical detection of lead(II) based on waste-biomass-derived carbon quantum dots@Zeolitic Imidazolate framework-8. Materials. 10.3390/ma16093378 37176266 10.3390/ma16093378 PMC10180021

[CR108] Li X et al (2023) Beer-derived nitrogen, phosphorus co-doped carbon quantum dots: Highly selective on–off-on fluorescent probes for the detection of ascorbic acid in fruits. Food Chem 409:135243 36584525 10.1016/j.foodchem.2022.135243

[CR48] Li Y-S et al (2024) Carbon quantum dots derived from camphor tree leaves biomass as a highly selective probe for Fe3 + sensing. Biomass Convers Biorefinery 14(20):26521–26531

[CR23] Li Z et al (2021) Green synthesis of carbon quantum dots from corn stalk shell by hydrothermal approach in near-critical water and applications in detecting and bioimaging. Microchem J 166:106250–106250

[CR24] Lv S et al (2022) Preparation and application of chitosan-based fluorescent probes. Analyst 147(21):4657–4673 36155993 10.1039/d2an01070d

[CR25] Lv S et al (2023) Aminophenol functionalized carbon quantum dots as fluorescent sensor for nitroalkenes. Microchem J 189:108569–108569

[CR60] Ma H et al (2023) Synthesis and enhancement of carbon quantum dots from Mopan persimmons for Fe3 + sensing and anti-counterfeiting applications. Chem Eng J 453:139906

[CR30] Manikandan V, Min SC (2023) Biofabrication of carbon quantum dots and their food packaging applications: a review. Food Sci Biotechnol 32(9):1159–1171 37362813 10.1007/s10068-023-01309-x PMC10290018

[CR92] Manjubaashini N, Bargavi P, Balakumar S (2024) Carbon quantum dots derived from agro waste biomass for pioneering bioanalysis and in vivo bioimaging. J Photochem Photobiol A 454:115702

[CR31] Mansi M, Gaurav S (2023) Synthesis and applications of carbon dots from waste biomass, in carbon dots in analytical chemistry, pp. 319–328. Elsevier

[CR21] Mathew S, Mathew B (2023) A review on the synthesis, properties, and applications of biomass derived carbon dots. Inorg Chem Commun 156:111223–111223

[CR115] Mathew S, Mathew B (2023) Biomass-derived carbon dots as a nanoswitch, logic gate operation, and electrochemical sensor for flavonoids. New J Chem 47(5):2383–2395

[CR84] Mohamed RMK et al (2023) Bifunctional ratiometric sensor based on highly fluorescent nitrogen and sulfur biomass-derived carbon nanodots fabricated from manufactured dairy product as a precursor. Spectrochim Acta Part A Mol Biomol Spectrosc 293:122444 10.1016/j.saa.2023.122444 36758366

[CR78] Mu L et al (2024) Simultaneous synthesis of carbon quantum dots and hydrothermal biochar from corn straw through hydrothermal treatment. Ind Crops Prod 219:119026

[CR32] Nagaraj M et al (2022) Detection of Fe3 + ions in aqueous environment using fluorescent carbon quantum dots synthesized from endosperm of Borassus flabellifer. Environ Res 212:113273–113273 35439456 10.1016/j.envres.2022.113273

[CR33] Osman MM, El-Shaheny R, Ibrahim FA (2024) Alfalfa biomass as a green source for the synthesis of N, S-CDs via microwave treatment. Application as a nano sensor for nifuroxazide in formulations and gastric juice. Anal Chim Acta 1319:342946–342946 39122268 10.1016/j.aca.2024.342946

[CR87] Padmapriya A et al (2023) Electrochemical sensor based on N,P–doped carbon quantum dots derived from the banana flower bract (Musa acuminata) biomass extract for selective and picomolar detection of dopamine. J Electroanal Chem 943:117609

[CR27] Pang Z et al (2022) Efficient ethanol solvothermal synthesis of high-performance nitrogen-doped carbon quantum dots from lignin for metal ion nanosensing and cell imaging. Ind Crops Prod 183:114957

[CR15] Pechnikova NA et al (2025) Carbon quantum dots in biomedical applications: advances, challenges, and future prospects. Aggregate 6(3):e707

[CR34] Pechnikova NA et al (2025) Carbon quantum dots in biomedical applications: advances, challenges, and future prospects. Aggregate 6(3):e707–e707

[CR35] Preethi M et al (2022) Potato starch derived N-doped carbon quantum dots as a fluorescent sensing tool for ascorbic acid. J Photochem Photobiol A 431:114009–114009

[CR82] Preethi M, Viswanathan C, Ponpandian N (2022) Fluorescence quenching mechanism of P-doped carbon quantum dots as fluorescent sensor for Cu2 + ions. Colloids Surf A 653:129942

[CR91] Qandeel NA et al (2023) Fast one-pot microwave-assisted green synthesis of highly fluorescent plant-inspired S,N-self-doped carbon quantum dots as a sensitive probe for the antiviral drug nitazoxanide and hemoglobin. Anal Chim Acta 1237:340592 36442950 10.1016/j.aca.2022.340592

[CR90] Qi H et al (2019) Biomass-derived nitrogen-doped carbon quantum dots: highly selective fluorescent probe for detecting Fe3 + ions and tetracyclines. J Colloid Interface Sci 539:332–341 30594008 10.1016/j.jcis.2018.12.047

[CR51] Qureashi A et al (2021) Biomass-derived carbon quantum dots: a novel and sustainable fluorescent ON–OFF–ON sensor for ferric ions. Anal Methods 13(40):4756–4766 34559168 10.1039/d1ay01112j

[CR36] Raikwar VR (2022) Synthesis and study of carbon quantum dots (CQDs) for enhancement of luminescence intensity of CQD@ LaPO4: Eu3 + nanocomposite. Mater Chem Phys 275:125277–125277

[CR50] Raja S et al (2024) Biomass-derived carbon quantum dot: on-off-on fluorescent sensor for rapid detection of multi-metal ions and green photocatalytic CO2 reduction in water. Biomass Convers Biorefinery 14(18):21925–21937

[CR86] Raju KS, Das GS, Tripathi KM (2024) Nitrogen-doped carbon quantum dots from biomass as a FRET-based sensing platform for the selective detection of H 2 O 2 and aspartic acid. RSC Sustain 2(1):223–232

[CR95] Reagen S et al (2021) Synthesis of highly near-infrared fluorescent graphene quantum dots using biomass-derived materials for in vitro cell imaging and metal ion detection. ACS Appl Mater Interfaces 13(37):43952–43962 34495635 10.1021/acsami.1c10533

[CR67] Ren H et al (2023) Turning Agroforestry waste into value-added fluorescent carbon quantum dots for effective detection of Fe3 + in an aqueous environment. ACS ES&T Eng 3:260–270

[CR70] Ren H et al (2024) Transforming bio-waste lignin into amine functionalized carbon quantum dots for selective detection of trace Cu2 + in aqueous system. Int J Biol Macromol 273:133118 38871106 10.1016/j.ijbiomac.2024.133118

[CR88] Sangubotla R, Won S, Kim J (2023) Boronic acid-modified fluorescent sensor using coffee biowaste-based carbon dots for the detection of dopamine. J Photochem Photobiol A 438:114542

[CR73] Santiago DLD et al (2025) Green synthesis of carbon quantum dots (CQDs) from beetroot by carbonization method and their application in metal ion sensing (Cu2+). MRS Adv 10:1602

[CR37] Selvaraju N et al (2022) Electron transfer behaviour of green synthesized carbon quantum dot sensor towards VOC and heavy metal ion sensing. Mater Sci Engineering: B 282:115792–115792

[CR17] Sharma A et al (2021) Upgrading of seafood waste as a carbon source: nano-world outlook. J Environ Chem Eng 9(6):106656

[CR100] Shi Y et al (2024) A novel sustainable biomass-based fluorescent probe for sensitive detection of salicylic acid in rice. Food Chem 434:137260 37713760 10.1016/j.foodchem.2023.137260

[CR118] Singh A et al (2023) Fabrication of FRET based nano sensor from biomass-derived fluorescent carbon quantum dots and naphthalimide for ratiometric detection of nitric oxide: To examine nitrite levels in meat samples. Anal Chim Acta 1270:341444 37311616 10.1016/j.aca.2023.341444

[CR53] Singh P et al (2023) Assessment of biomass-derived carbon dots as highly sensitive and selective templates for the sensing of hazardous ions. Nanoscale 15(40):16241–16267 37439261 10.1039/d3nr01966g

[CR38] Sun X et al (2021) Construction of ratiometric fluorescence MIPs probe for selective detection of tetracycline based on passion fruit peel carbon dots and europium. Microchim Acta 188(9):297–297 10.1007/s00604-021-04929-4 34401956

[CR101] Sun X-H et al (2023) One-pot hydrothermal method preparation of cerium–nitrogen-codoped carbon quantum dots from waste longan nucleus as a fluorescent sensor for sensing drug rifampicin. ACS Omega 8(38):34859–34867 37780005 10.1021/acsomega.3c04242 PMC10536864

[CR85] Tao X et al (2022) Designing of biomass-derived carbon quantum dots@polyvinyl alcohol film with excellent fluorescent performance and pH-responsiveness for intelligent detection. Chem Eng J 443:136442

[CR14] Teli S, Soni S, Agarwal S (2025) Harnessing bioinspired carbon quantum dots: a six-year odyssey in synthesis and catalytic revolution. Crit Rev Solid State Mater Sci 51:1–62

[CR75] Tony Elizabeth A et al (2024) Morinda coreia fruits derived green-emissive nitrogen-doped carbon quantum dots: selective and sensitive detection of ferric ions from water. Inorg Chem Commun 164:112390

[CR19] Tripathi VK et al (2023) N-doped graphene nanosheets-based optical nano switch for the selective detection of guanine and Pb 2+. RSC Sustain 1(9):2319–2327

[CR129] Universidade de Santiago de Compostela (2024) ICP-MS section: detection limits. Unidade de Análise Instrumental. https://assets.usc.gal/en/services/area/research-infrastructures/services-and-equipment/instrumental-analysis/icp-ms-section. Accessed 24 Feb 2026

[CR39] Vyas T, Gogoi M, Joshi A (2023) Fluorescent fiber-optic device sensor based on carbon quantum dot (CQD) thin films for dye detection in water resources. Analyst 148(20):5178–5189 37721153 10.1039/d3an01343j

[CR113] Wang J et al (2024) A dual-emitting fluoroprobe fabricated by aloe leaf-based N-doped carbon quantum dots and copper nanoclusters for nitenpyram detection in waters by virtue of inner filter effect and static quenching principles. Anal Chim Acta 1289:342182 38245198 10.1016/j.aca.2023.342182

[CR120] Wang P et al (2022) Loquat fruit-based carbonquantumdots as an ON-OFF probe for fluorescent assay of MnO4 – in waters based on the joint action of inner filter effect and static quenching. Microchem J 178:107374

[CR71] Wang S et al (2023) Preparation of multicolor biomass carbon dots based on solvent control and their application in Cr(VI) detection and advanced anti-counterfeiting. ACS Omega 8(7):6550–6558 36844529 10.1021/acsomega.2c06942 PMC9948216

[CR111] Wang X et al (2023) A fluorescence visual detection for glyphosine based on a biomass carbon quantum dot paper-based sensor. New J Chem 47(22):10696–10705

[CR123] Wang Y et al (2021) Hydrothermal synthesis of nitrogen-doped carbon quantum dots from lignin for formaldehyde determination. RSC Adv 11(47):29178–29185 35479568 10.1039/d1ra05370a PMC9040886

[CR110] Wei X et al (2023) Carbon quantum dot/chitosan-derived hydrogels with photo-stress-pH multiresponsiveness for wearable sensors. Macromol Rapid Commun 44(8):2200928 10.1002/marc.202200928 36786588

[CR96] Wu C et al (2023) Sensitive and smartphone-assisted visual detection of oxytetracycline by a ratiometric fluorescence sensor based on nitrogen-doped carbon quantum dots from banana peel cooperating with europium. Microchem J 194:109283

[CR122] Wu Z et al (2022) Flexible all-biomass gas sensor based on doped carbon quantum dots/nonwoven cotton with discriminative function. Cellulose 29(10):5817–5832

[CR68] Xia L et al (2022) Sustainable and green synthesis of waste-biomass-derived carbon dots for parallel and semi-quantitative visual detection of Cr(VI) and Fe3+. Molecules. 10.3390/molecules27041258 35209046 10.3390/molecules27041258 PMC8876948

[CR74] Yang J, Guo Z, Yue X (2022) Preparation of carbon quantum dots from corn straw and their application in Cu2 + detection. BioResources, 17(1)

[CR16] Yang X et al (2024) A true biomass standout: preparation and application of biomass-derived carbon quantum dots. BioResources 19(3)

[CR40] Yang X et al (2024) Carbon quantum dots derived from rice straw doped with N and S and its nanocomposites with hydroxypropyl cellulose nanocomposite. Int J Biol Macromol 278:134925–134925 39217044 10.1016/j.ijbiomac.2024.134925

[CR81] Yan J et al (2021) Highly fluorescent N-doped carbon quantum dots derived from bamboo stems for selective detection of Fe3 + ions in biological systems. J Biomed Nanotechnol 17(2):312–321 33785101 10.1166/jbn.2021.3034

[CR103] Yin C, Chen L, Niu N (2021) Nitrogen-doped carbon quantum dots fabricated from cellulolytic enzyme lignin and its application to the determination of cytochrome c and trypsin. Anal Bioanal Chem 413(20):5239–5249 34212211 10.1007/s00216-021-03496-0

[CR80] Yin Y et al (2024) Carbon quantum dot (CQD)-based composite fluorescent hydrogel for the isolation and determination of iron (III). Anal Lett 57(10):1595–1610

[CR41] Yong C et al (2023) Mechanistic regulation of gram-scale synthesis of triple emission cyanobacteria-based carbon dots and visual ratiometric sensing applications. Appl Surf Sci 623:157049–157049

[CR42] Zhang B et al (2024) Green synthesis of biomass-derived porous carbon for electrochemical detection of heavy metal ions: methods, properties, and applications. J Environ Chem Eng 12(5):113903–113903

[CR43] Zhang D et al (2023) Red-to-blue colorimetric probe based on biomass carbon dots for smartphone-integrated optosensing of Cu (II) and L-cysteine. Spectrochim Acta Part A Mol Biomol Spectrosc 290:122285–122285 10.1016/j.saa.2022.122285 36592594

[CR22] Zhang L et al (2022) A review on carbon quantum dots: synthesis, photoluminescence mechanisms and applications. Luminescence 37(10):1612–1638 35906748 10.1002/bio.4351

[CR119] Zhang Q et al (2022) Targeted ginkgo kernel biomass precursor using eco-friendly synthesis of efficient carbon quantum dots for detection of trace nitrite ions and cell imaging. Inorg Chem Commun 140:109442

[CR44] Zhang S et al (2023) One-pot solvothermal preparation of triple-emission N, Cl doped carbon quantum dots from waste traditional Chinese medicines as a fluorescent sensor for sensing water and Cr (Ⅵ). Colloids Surf A 669:131471–131471

[CR109] Zhao Q et al (2023) One-pot synthesis of environmentally-friendly carbon quantum dots for on-off rapid fluorescent sensing of folic acid, Fe3+, and Ca2+. J Lumin 263:120091

[CR116] Zhao Y et al (2024) Green synthesized carbon quantum dots as chemiluminescence sensor for sulfanilamide detection. Dyes Pigm 225:112087

[CR66] Zhong L et al (2025) Dual roles of carbon quantum dots from green carbon sources: a fluorescence sensor for Fe3 + ions, UV and high-energy blue light screening. Nanomaterials. 10.3390/nano15060436 40137609 10.3390/nano15060436 PMC11945768

[CR62] Zhou C et al (2021) Facile and high-yield synthesis of N-doped carbon quantum dots from biomass quinoa saponin for the detection of Co2+. J Anal Methods Chem 2021(1):9732364 34976427 10.1155/2021/9732364 PMC8718314

[CR45] Zhou C et al (2023) Green synthetic carbon quantum dots based on waste tobacco leaves and its application to detecting borax content in flour and its products. J Mol Struct 1278:134959–134959

[CR127] Zhou R et al (2022) The self-nitrogen-doped carbon quantum dots derived from Morus alba L. leaves for the rapid determination of tetracycline. Ind Crops Prod 188:115705–115705

[CR105] Zhu J et al (2021) Green preparation of carbon dots from plum as a ratiometric fluorescent probe for detection of doxorubicin. Opt Mater 114:110941

[CR134] Eliboev, Ilyos, et al. "Advancing analytical chemistry with carbon quantum dots: a comprehensive review." Analytical Methods 17.13 (2025): 2627-2649. 10.1039/d4ay02237h 40104848 10.1039/d4ay02237h

[CR26] Zhu L et al (2021) Sustainable synthesis of bright green fluorescent carbon quantum dots from lignin for highly sensitive detection of Fe3 + ions. Appl Surf Sci 565:150526

[CR77] Zhu L, Shen D, Hong K, Luo (2022) Triple-emission nitrogen and boron co-doped carbon quantum dots from lignin: highly fluorescent sensing platform for detection of hexavalent chromium ions. J Colloid Interface Sci 617:557–567 35303639 10.1016/j.jcis.2022.03.039

[CR102] Zhu Y et al (2023) Coffee grounds-derived carbon quantum dots as peroxidase mimetics for colorimetric and fluorometric detection of ascorbic acid. Food Chem 429:136957 37499505 10.1016/j.foodchem.2023.136957

[CR135] Allambergenova, Farida, et al. "Synthesis of carbon dots based on chitosan and melamine and their application in detecting vanadate (V) anions." Chinese Journal of Analytical Chemistry 53.7 (2025): 100538.

[CR136] Kiryigitova, Sevara, et al. "Application of carbon dots synthesized from chitosan and dithizone for the detection of mercury (II) ions." Chinese Journal of Analytical Chemistry (2026): 100710.

